# RNA-binding protein PCBP2 regulates pancreatic **β** cell function and adaptation to glucose

**DOI:** 10.1172/JCI172436

**Published:** 2024-06-17

**Authors:** Matthew W. Haemmerle, Andrea V. Scota, Mina Khosravifar, Matthew J. Varney, Sabyasachi Sen, Austin L. Good, Xiaodun Yang, Kristen L. Wells, Lori Sussel, Andrea V. Rozo, Nicolai M. Doliba, Louis R. Ghanem, Doris A. Stoffers

**Affiliations:** 1 Institute for Diabetes, Obesity, and Metabolism and Department of Medicine, Perelman School of Medicine at the University of Pennsylvania, Philadelphia, Pennsylvania.; 2 Department of Pediatrics and; 3 Department of Cell & Developmental Biology, and; 4 Barbara Davis Center for Diabetes, University of Colorado Anschutz Medical Campus, Aurora, Colorado, USA.; 5 Division of Gastroenterology, Hepatology and Nutrition Division, Children’s Hospital of Philadelphia, Philadelphia, Pennsylvania, USA.; 6 Department of Pediatrics, Perelman School of Medicine at the University of Pennsylvania, Philadelphia, Pennsylvania, USA.

**Keywords:** Endocrinology, Beta cells, Diabetes, Glucose metabolism

## Abstract

Glucose plays a key role in shaping pancreatic β cell function. Thus, deciphering the mechanisms by which this nutrient stimulates β cells holds therapeutic promise for combating β cell failure in type 2 diabetes (T2D). β Cells respond to hyperglycemia in part by rewiring their mRNA metabolism, yet the mechanisms governing these changes remain poorly understood. Here, we identify a requirement for the RNA-binding protein PCBP2 in maintaining β cell function basally and during sustained hyperglycemic challenge. PCBP2 was induced in primary mouse islets incubated with elevated glucose and was required to adapt insulin secretion. Transcriptomic analysis of primary *Pcbp2-*deficient β cells revealed impacts on basal and glucose-regulated mRNAs encoding core components of the insulin secretory pathway. Accordingly, *Pcbp2-*deficient β cells exhibited defects in calcium flux, insulin granule ultrastructure and exocytosis, and the amplification pathway of insulin secretion. Further, PCBP2 was induced by glucose in primary human islets, was downregulated in islets from T2D donors, and impacted genes commonly altered in islets from donors with T2D and linked to single-nucleotide polymorphisms associated with T2D. Thus, these findings establish a paradigm for PCBP2 in governing basal and glucose-adaptive gene programs critical for shaping the functional state of β cells.

## Abstract 2

RNA-binding protein PCBP2 programs the functional capacity of pancreatic islet β cells during acute and sustained high glucose challenge.

## Graphical abstract

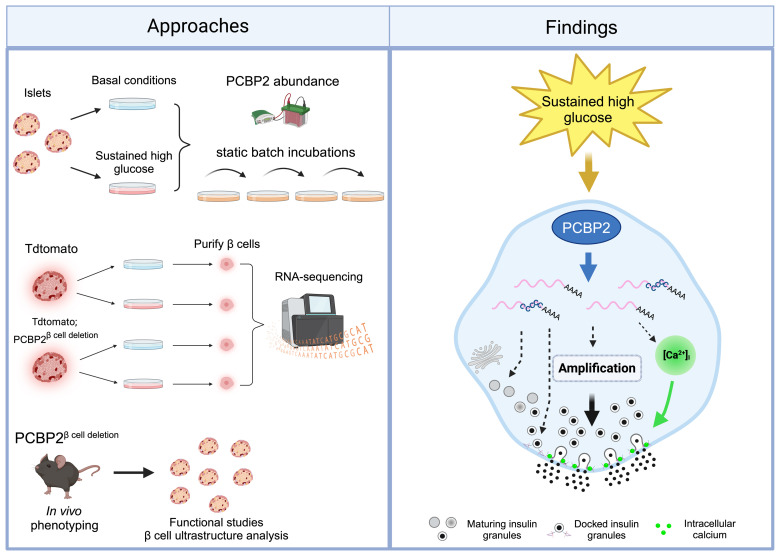

## Introduction

Glucose uptake and metabolism are the most physiologically important driver of insulin release from pancreatic islet β cells, a highly specialized class of endocrine cells that secrete this hormone in the regulation of organismal blood glucose homeostasis (1). It is well known that glucose has pleiotropic effects on β cell physiology depending on the timing and severity of exposure. Acute and short-term hyperglycemia challenges augment the functional status of β cells on multiple levels by enhancing the flux of glucose metabolism, the expression of machinery critical for insulin secretion, and the responsiveness of β cells to metabolic stimulation (2, 3). Collectively, these alterations serve to magnify insulin secretion to meet increased insulin secretion demands in response to increased metabolic stimulation. In contrast to these stimulatory effects, chronic high glucose exposure paradoxically impairs the ability of β cells to respond to and to secrete appropriate amounts of insulin through combinatorial actions that promote metabolic dysfunction, downregulation of β cell functional and identity genes, and induction of stress-response and apoptotic gene programs (2). The central role of β cell failure in diabetes pathogenesis has driven strong interest in understanding how β cells respond to and adapt to different conditions of glucose challenge in the hope of identifying therapeutic interventions (4). However, despite the major inroads that have been made in clarifying the mechanisms that shape these processes, the complex and diverse impact glucose exerts on the β cell suggests that much remains to be discovered regarding the mechanisms that adapt β cells to high-glucose challenges.

β Cells can adapt to high-glucose challenges in part by reshaping their mRNA metabolism, thereby providing rapid fine-tuning of gene expression to preserve cellular homeostasis (5, 6). For instance, the pre-proinsulin mRNA is under complex levels of posttranscriptional regulation by glucose, and its stability and translation are enhanced in response to acute high-glucose challenge (7, 8). Similarly, the stability and translation of mRNAs encoding core components required for insulin secretion and β cell survival can be modulated by glucose (9–11). Glucose also alters the splicing of certain transcripts encoding modulators of insulin secretion (12–14). Collectively, these observations support the notion that glucose is a powerful regulator of posttranscriptional gene regulation in the β cell and influences the functional state of β cells.

RNA-binding proteins (RBPs) are master regulators of posttranscriptional gene regulation and modulate all aspects of the life cycles of mRNAs (15). These factors typically function by interacting with sequence-specific motifs embedded in target mRNAs, which can serve as focal points to regulate different aspects of mRNA processing, such as splicing, 3′-UTR processing, mRNA stability, and translation (16). RBPs also have the capacity to reprogram cells in response to extracellular stimuli by reshaping mRNA metabolism to facilitate rapid changes in gene expression (17). Despite the powerful role RBPs have in mRNA processing and the hundreds of RBPs known to be expressed in the β cell (13), their importance to β cell biology has only recently gained traction (5, 18). Several RBPs have been implicated in reshaping the metabolism of select transcripts critical in enhancing insulin secretion in response to high-glucose challenge (5, 6). However, the impact of RBPs on the posttranscriptional landscape on a global scale and their relevance to primary β cells remain less well described.

The poly(C)-binding proteins (PCBPs) are a functionally heterogeneous family of RBPs composed of 5 members (PCBP1–PCBP4 and HRNPK) that are defined by their shared binding specificity for cytosine-rich sequences (19). The PCBPs have been extensively shown to govern a variety of posttranscriptional controls and have been implicated in shaping a multitude of cellular events (19). In addition, these proteins can serve as cytosolic iron chaperones and play integral roles in the transport, delivery, and storage of intracellular iron (20). Despite the critical roles these factors play in cellular homeostasis, an understanding of the impact of the PCBPs in the β cell remains limited, and whether they impact the functional state of β cells is unknown (21, 22). While mining publicly available data sets, we identified PCBP2 as a putatively glucose-regulated protein from a mass spectrometry study characterizing glucose-regulated proteins in human islets (23), raising the possibility that PCBP2 could modulate important glucose effects in the β cell.

Here, we demonstrate a critical requirement for the RBP PCBP2 in the β cell, revealing roles for this factor in maintaining the functional state of the β cell basally and during adaptation to a sustained hyperglycemic challenge. PCBP2 was upregulated in murine islets incubated in high glucose and was required for the adaptive insulin secretory response. Transcriptomic and motif enrichment analysis of FACS-purified, *Pcbp2-*null β cells under basal and high-glucose challenge implicated PCBP2 in shaping the abundance of mRNAs necessary to sustain and to adapt components of the insulin secretory pathway. Mechanistic studies revealed that PCBP2 impacted the β cell transcriptome in part by binding and stabilizing its regulated mRNAs. Accordingly, *Pcbp2* deficiency impaired insulin secretion, owing to defects in insulin production, insulin granule ultrastructure and docking, calcium flux, and the amplification pathway of insulin secretion. In primary human islets, PCBP2 was upregulated by high-glucose challenge while also downregulated in islets from donors with type 2 diabetes (T2D), suggesting roles for this factor in human islet glucose adaptation and T2D pathophysiology. Indeed, we found that the PCBP2-regulated genes overlapped with those altered in islets from human T2D and linked to single-nucleotide polymorphisms associated with T2D. Together, these studies position PCBP2 as a core posttranscriptional determinant of the functional state of β cells.

## Results

### PCBP2 is posttranscriptionally upregulated in islets during high-glucose incubation and regulates glucose-adaptive insulin secretion.

**Figure 1:**
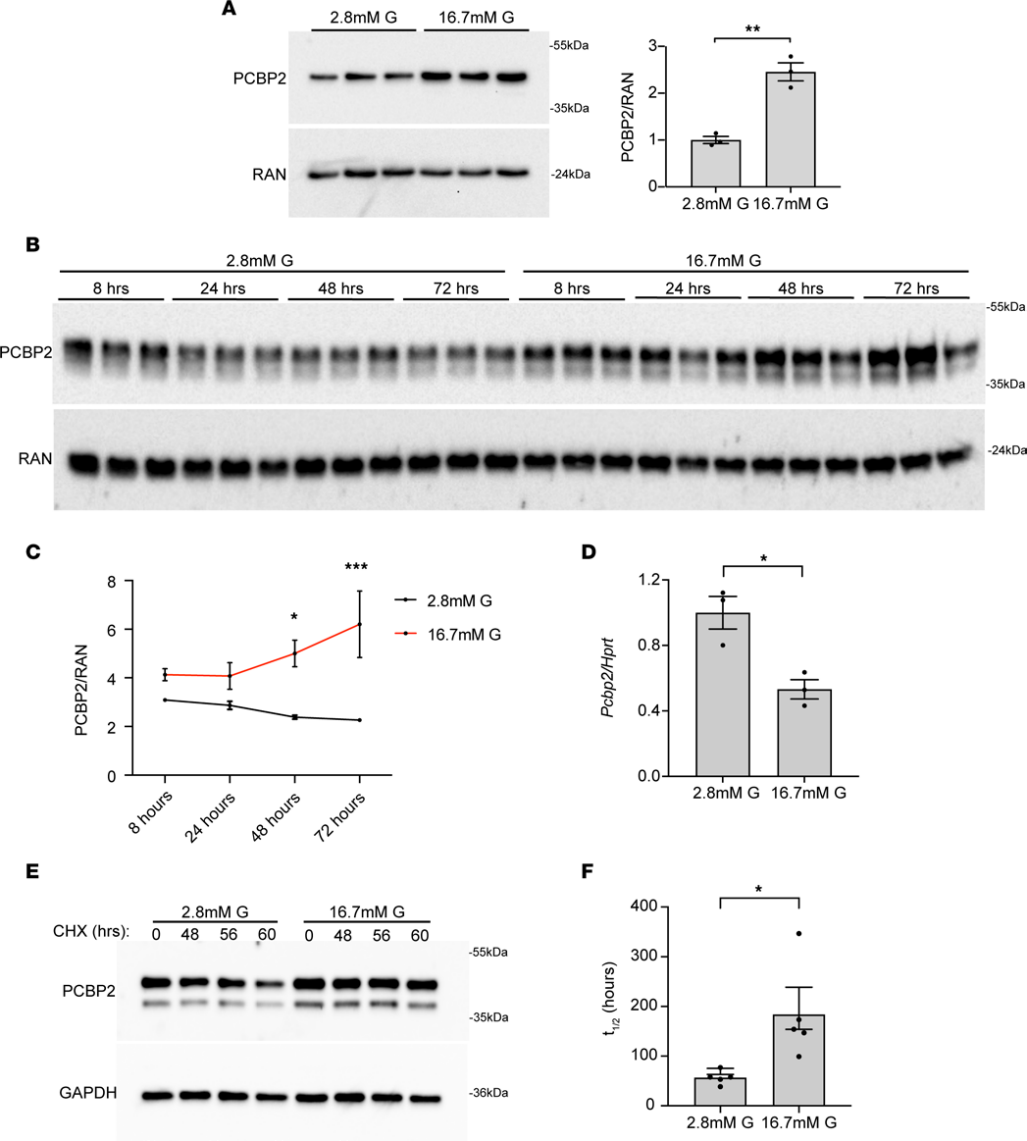
PCBP2 is posttranscriptionally upregulated in islets incubated with high glucose. (**A**–**C**) Western blot and quantification showing PCBP2 levels in mouse islets from 7- to 9-week-old wild-type male mice incubated in 2.8 mM or 16.7 mM glucose for 48 hours (*n* = 3) (**A**) and 2.8 mM or 16.7 mM glucose for 24, 48, and 72 hours (*n* = 3) (**B** and **C**). (**D**) Decreased *Pcbp2* transcript levels in mouse islets from 7- to 8-week-old wild-type male mice incubated with 16.7 mM glucose for 48 hours (*n* = 3). (**E**) Representative Western blot showing reduction in PCBP2 levels at indicated times after addition of cycloheximide (CHX) to mouse islets from 8- to 10-week-old wild-type male mice. CHX was added after culturing for 72 hours in 2.8 mM or 16.7 mM glucose. (**F**) Quantification of PCBP2 *t*_1/2_ in islets incubated with 2.8 mM or 16.7 mM glucose (*n* = 5). *P < 0.05, **P < 0.01 by Student’s 2-tailed *t* test (**A**, **D**, and **F**); and *P < 0.05, ***P < 0.001 by 2-way repeated-measures ANOVA with Holm-Šidák post hoc test (**C**).

Based on mining of a publicly available human islet proteomic data set, we raised the RBP PCBP2 as a potentially glucose-induced protein (23). To determine whether PCBP2 levels could be modulated by glucose and to provide a platform for further study, we examined PCBP2 in murine, wild-type islets exposed to elevated levels of glucose. Interestingly, we observed that PCBP2 increased about 2.5-fold in these stimulated islets as compared with their low-glucose-treated counterparts (Figure 1A). A time course experiment revealed that high-glucose culture conditions increased PCBP2 in a time-dependent manner in mouse islets, with a maximal approximately 3-fold PCBP2 induction occurring at 72 hours of culture (Figure 1, B and C). PCBP2 levels did not further increase in response to higher levels of glucose, and dosing islets with a range of physiologically relevant levels of glucose also confirmed the induction of PCBP2 as glucose dependent (Supplemental Figure 1, A and B; supplemental material available online with this article; https://doi.org/10.1172/JCI172436DS1). Under low-glucose conditions, PCBP2 expression was lower in insulin-positive cells than in non-β cells, whereas expression was similar across all islet endocrine cell types under high-glucose conditions (Supplemental Figure 2A), suggesting that glucose regulation of PCBP2 levels specifically occurs in the β cell lineage. Further, PCBP2 subcellular localization did not appear to change under different glucose conditions (Supplemental Figure 2B).

We next considered the mechanism by which glucose modulates PCBP2. Despite increased PCBP2 during high-glucose challenge, the *Pcbp2* transcript was decreased (Figure 1D). The decoupling between protein and mRNA changes pointed to glucose posttranscriptionally modulating PCBP2 abundance. To determine whether glucose influences PCBP2 protein stability, we treated murine islets preincubated with low or high glucose with the translational inhibitor cycloheximide and measured the turnover of PCBP2 protein in both groups. Culture in high glucose decreased the rate of decay of PCBP2 and increased the half-life of PCBP2 from about 50 hours to about 200 hours, indicative of increased stability (Figure 1, E and F).

**Figure 2:**
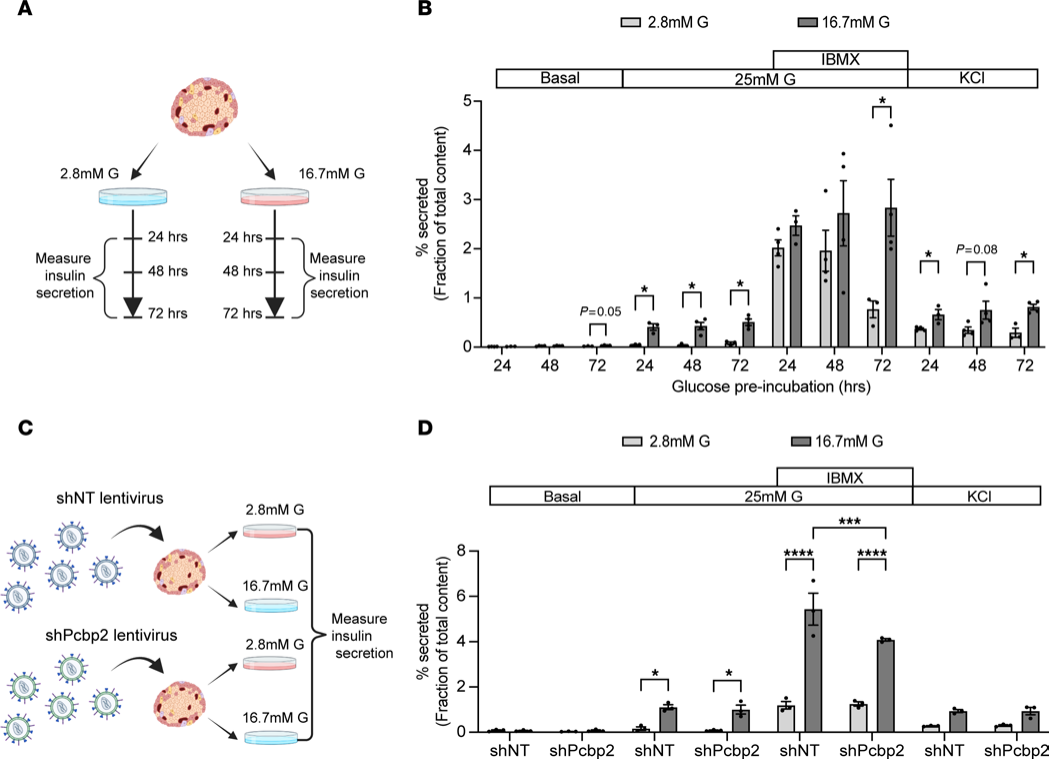
β Cell–specific depletion of *Pcbp2* blunts IBMX augmentation of insulin secretion during glucose adaptation. (**A**) Schematic of batch incubation time course to examine changes in β cell function during high glucose exposure conditions leading to maximal PCBP2 abundance. (**B**) Static insulin secretion profiles of islets from 13- to 17-week-old wild-type male mice preincubated with 2.8 mM or 16.7 mM glucose for indicated time periods and stimulated with 2.8 mM glucose, 25 mM glucose ± 0.1 mM IBMX, and 30 mM KCl for 45 minutes (*n* = 3–4). (**C**) Schematic of batch incubation experimental design to examine the functional requirement of PCBP2 in glucose-adaptive insulin secretion. (**D**) Static insulin secretion profiles of transduced islets with non-targeting shRNA (shNT) or shRNA targeting *Pcbp2* (shPcbp2) preincubated with 2.8 mM or 16.7 mM glucose. Islets were acutely stimulated with 2.8 mM glucose, 25 mM glucose ± 0.1 mM IBMX, and 30 mM KCl for 45 minutes (*n* = 3). *P < 0.05 by Student’s 2-tailed *t* test (**B**); and *P < 0.05, ***P < 0.001, ****P < 0.0001 by 2-way repeated-measures ANOVA with Holm-Šidák post hoc test (**D**).

Glucose can augment or impair β cell insulin secretion depending on the timing and severity of exposure (2); therefore, we sought to define how high-glucose culture conditions driving increased PCBP2 levels impacted β cell function. We assessed insulin secretion of wild-type islets that had been preincubated with low or high glucose for 24, 48, and 72 hours (Figure 2A). The islets were then acutely challenged with increasing glucose concentrations to assess overall insulin secretion, IBMX to assess cAMP-mediated amplification of insulin secretion, and potassium chloride (KCl) to examine release of the readily releasable pool of insulin granules. Islets preincubated with high glucose generally exhibited augmented insulin secretion both basally and in response to acute stimuli, and this augmentation was similar in magnitude across 24, 48, and 72 hours of preincubation (Figure 2B and Supplemental Figure 3A). Insulin secretion was amplified more than 10-fold in response to 25 mM glucose challenge in islets pretreated with high versus low glucose. Similarly, KCl stimulation elicited a greater than 2.5-fold insulin secretion response in high- versus low-glucose-precultured islets, and this response was sustained across each preincubation time point. The IBMX response diminished over time in islets preincubated with low glucose, but was maintained in islets pretreated with high glucose, suggesting that high glucose supports β cell processes necessary for cAMP-mediated insulin secretion. Insulin content was also increased in islets precultured with high glucose (Supplemental Figure 3B).

To examine the role of PCBP2 in the augmentation of insulin secretion during sustained high-glucose challenge, we employed a lentiviral construct driving mCherry and a non-targeting shRNA or a shRNA targeting *Pcbp2* from the rat insulin promoter to allow for β cell–specific depletion of *Pcbp2* in primary murine islets (24) (Supplemental Figure 4, A and B). To determine how depleting *Pcbp2* impacted glucose adaptation of insulin secretion, insulin secretion was measured from transduced islets preincubated with low or high glucose for 72 hours (Figure 2C). As expected, insulin secretion was amplified more than 4-fold in response to acute stimulation with 25 mM glucose, IBMX, and KCl from control islets that had been preincubated with high glucose (Figure 2D and Supplemental Figure 5). Islets with shRNA-mediated depletion of PCBP2 and pretreated with high glucose exhibited a similar augmentation of insulin secretion by 25 mM glucose and KCl; however, the augmented IBMX insulin secretory response was diminished. By contrast, glucagon secretion was normal during acute IBMX stimulation of *Pcbp2*-deficient islets (Supplemental Figure 6, A and B). Altogether, these results indicate that high-glucose culture conditions promoting increased PCBP2 augment β cell stimulus-coupled insulin secretion and that *Pcbp2* is required for glucose adaptation of the amplification pathway of insulin secretion.

### RNA sequencing identifies glucose-regulated gene programs in β cells.

**Figure 3:**
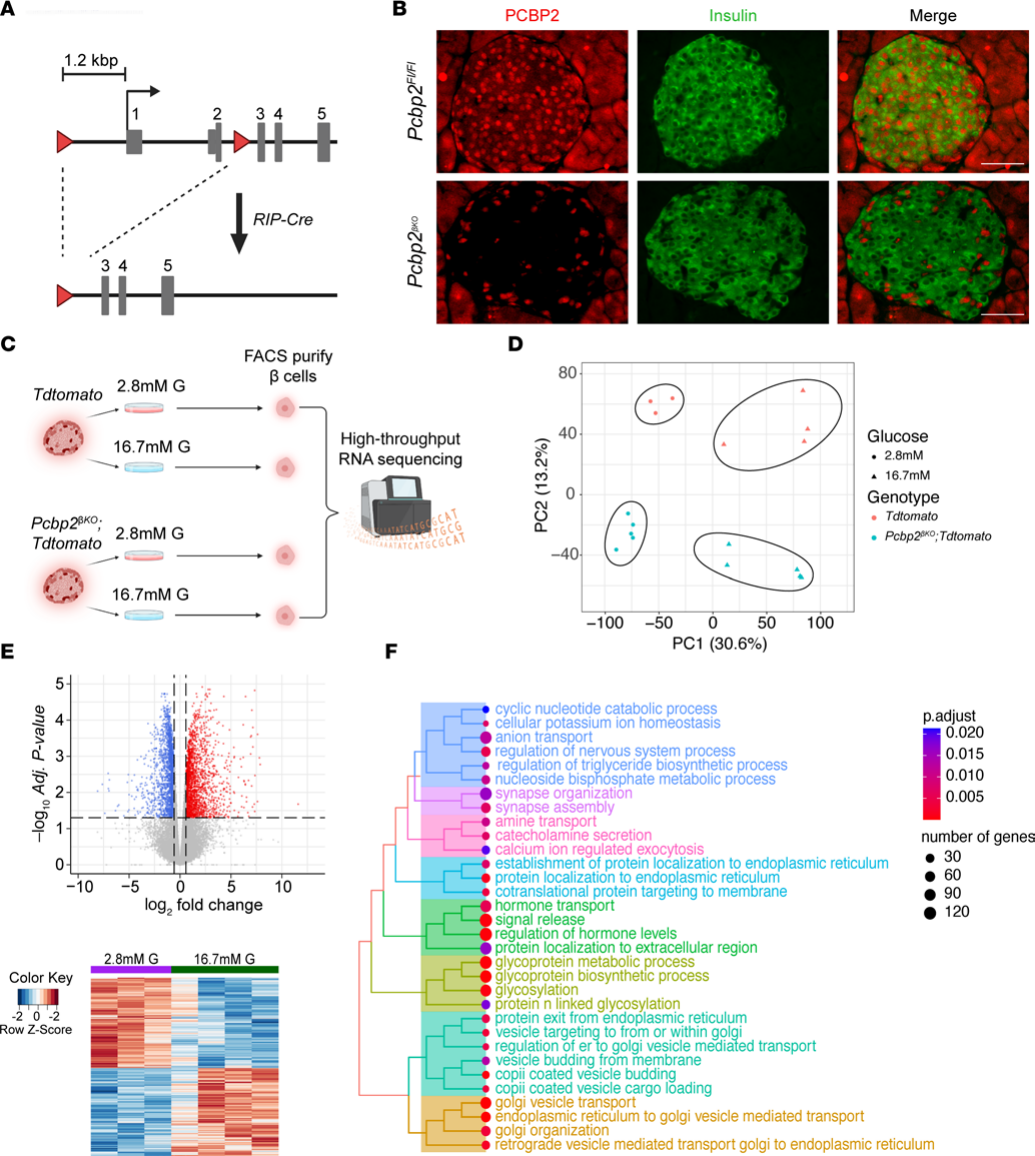
RNA sequencing of FACS-purified β cells during high-glucose incubation identifies glucose-regulated gene programs. (**A**) Model of Cre-mediated recombination of the *Pcbp2* conditional allele. The top diagram represents the *Pcbp2* locus (boxes represent UTRs, bars represent protein coding exons, and arrow represents transcriptional start site) with inserted *loxP* sites (red triangles). The bottom diagram represents the *Pcbp2* locus following *RIP-Cre*–mediated recombination and remaining *loxP* site (red triangle). (**B**) Coimmunofluorescent staining of PCBP2 and insulin on adult islets from 7- to 8-week-old *Pcbp2^fl/fl^* and *Pcbp2*^βKO^ mice showing efficient deletion of PCBP2 in β cells (scale bars: 50 μm). (**C**) Schematic showing strategy to isolate control and *Pcbp2-*deficient β cells cultured with high-glucose culture conditions promoting maximal PCBP2 levels for transcriptomic analysis. (**D**) Principal component analysis (PCA) of RNA-sequenced samples showing glucose treatment and genotype drive separation and clustering of samples (*n* = 3–5 per group). (**E**) Volcano plot (top) and heatmap (bottom) showing genes with transcript levels significantly altered by high-glucose challenge in control β cells. (**F**) Heatmap plot of terms overrepresented in the processes upregulated by glucose in control β cells.

We next sought to determine the molecular changes to the β cell during stimulation in high-glucose culture conditions. To address this question, we first crossed a RIP-Cre β cell–specific deleter strain with mice harboring the Cre-inducible *Rosa26^tdTomato^* reporter allele to selectively label the β cell lineage (termed *tdTomato*), providing the means to purify β cells that have undergone Cre-mediated recombination (Supplemental Figure 7, A and B). Islets isolated from *tdTomato* mice were then incubated in low and high glucose for 72 hours to maximally induce PCBP2 abundance followed by FACS enrichment of the tdTomato^+^ cell populations and RNA sequencing. In tandem, RIP-Cre mice were crossed with mice carrying *loxP Pcbp2* alleles (termed *Pcbp2*^βKO^) (25), resulting in efficient (~80%) depletion of PCBP2 specifically in β cells as determined by coimmunofluorescence for PCBP2 and insulin in adult islets (Figure 3, A and B). *Pcbp2*^βKO^ mice were then crossed with *tdTomato* mice (termed *Pcbp2*^βKO^ *tdTomato*), and islets from *Pcbp2*^βKO^ *tdTomato* mice were subjected to the same glucose exposure paradigm, tdTomato^+^ cell purification, and RNA sequencing strategy as that of islets from *tdTomato* mice (Figure 3C).

Principal component analysis (PCA) of RNA-sequenced samples revealed that glucose culture conditions and genotype accounted for the first and second highest proportions of overall sample variation, respectively, and that our replicates clustered together according to these experimental conditions (Figure 3D). To define the molecular changes resulting from stimulating β cells with high glucose, we performed differential gene expression analysis of control β cells treated with high glucose. Consistent with our PCA analysis, we found a substantial number of genes that were altered in response to high glucose exposure, with 1,981 and 1,933 genes down- and upregulated, respectively (Figure 3E and Supplemental Table 1). To comprehensively define the molecular pathways affected by exposing β cells to glucose, we performed gene set enrichment analysis (GSEA), a method that determines whether sets of functionally related genes show linked directionality changes between 2 conditions (26). Hierarchical clustering of upregulated pathway terms revealed an abundance of processes with terms related to hormone production and protein production and secretion (Figure 3F), consistent with the enhanced insulin secretion observed after preincubation with high glucose in our batch incubation analysis (Figure 2B). In contrast, downregulated processes were involved in RNA processing and posttranscriptional gene regulation (Supplemental Figure 7C), which may be a novel feature of β cell adaptation to glucose. Thus, our RNA sequencing approach captured glucose-regulated features in the β cell.

### PCBP2 impacts glucose-dependent and glucose-independent gene programs.

**Figure 4:**
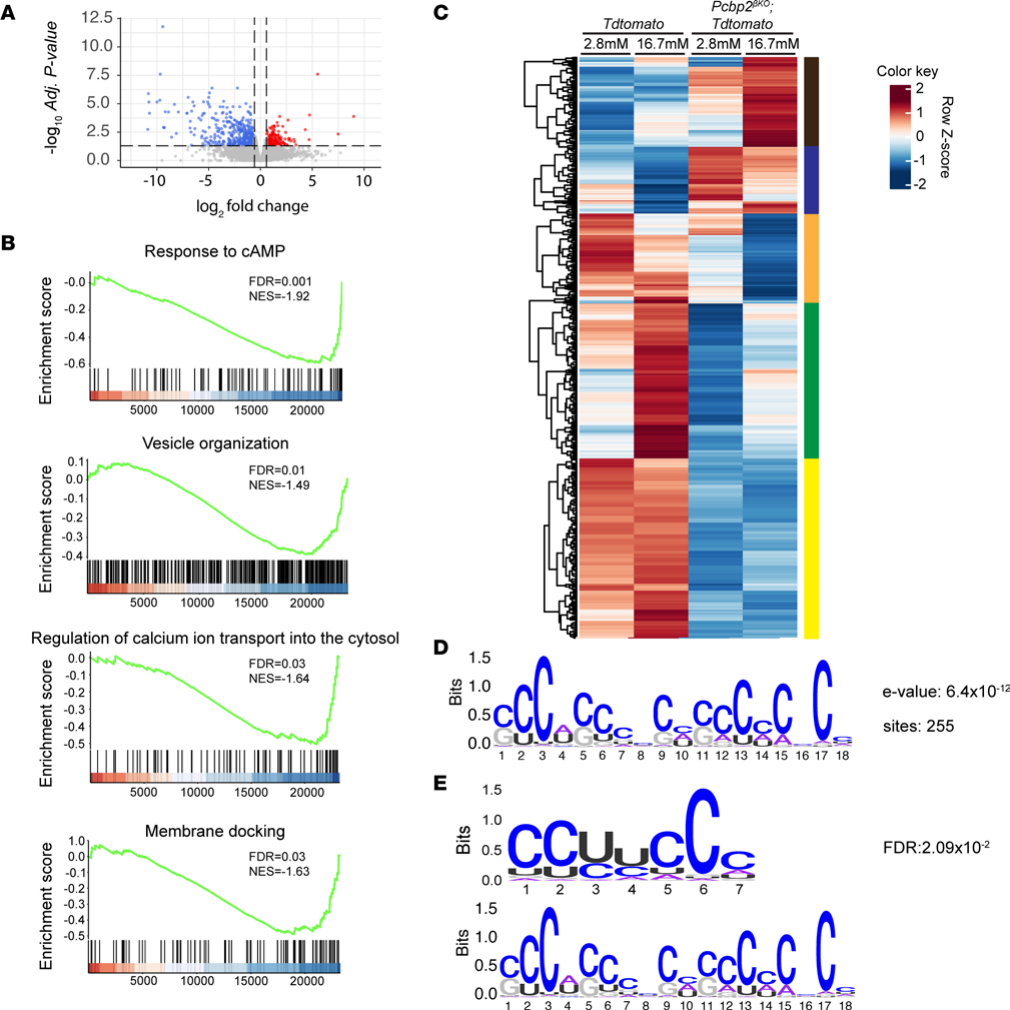
PCBP2 impacts sets of mRNAs encoding proteins required for sustaining and adapting β cell function. (**A**) Volcano plot showing genes with significantly altered mRNA levels between control and mutant β cells during high-glucose challenge. (**B**) GSEA plots showing pathways implicated in governing β cell function downregulated in mutant β cells during high-glucose preincubation. (**C**) Clustering analysis of PCBP2-regulated genes across control and mutant β cells under low- and high-glucose conditions. (**D**) Enriched motif found by MEME motif discovery in the 3′-UTRs of transcripts altered with *Pcbp2* deficiency during hyperglycemia. (**E**) Top match for aligned motifs corresponding to a known PCBP2 binding motif.

Given that glucose modulates PCBP2 (Figure 1, A and B), we next investigated whether PCBP2 played a role in shaping the transcriptomic response of β cells to glucose. We first compared the transcriptomes of control and *Pcbp2-*deficient β cells during high-glucose induction of maximal PCBP2 abundance, which revealed significant changes in the transcript abundance of 603 genes (Figure 4A and Supplemental Table 2), of which 439 and 164 exhibited decreased and increased transcript levels, respectively. GSEA identified pathways implicated in regulating key steps in insulin secretion that were downregulated in mutant β cells under high-glucose incubation, including cAMP responsiveness, vesicle organization, regulation of calcium ion transport, synapse organization and function, and membrane docking (Figure 4B and Supplemental Figure 8, A–C). In contrast, upregulated processes were involved in cellular replication (Supplemental Figure 9), which may signify a role for PCBP2 in shaping β cell replication.

To determine whether the PCBP2-regulated genes were also regulated by glucose, we compared the expression of these genes in control and mutant across low- and high-glucose preincubation conditions. Unbiased hierarchal clustering across these conditions raised 5 clusters of genes with distinct patterns of gene regulation (Figure 4C and Supplemental Figure 10, A–E). We identified a large cluster of genes (yellow cluster) that were unaffected by glucose treatment in control β cells but were downregulated irrespective of glucose treatment with *Pcbp2* deficiency. Genes mapping to this module have links to cAMP production and signaling (*Gpr26*, *Cftr*, *Hcn1*, *Adgrg6*, *Adgrd1*, *Adgrf1*, *Adgrf5*), synapse formation and activity (*Syt10*, *Kirrel3*, *Pdzrn3*, *Syndig1*, *Cast*), insulin secretory vesicles (*Rph3a*, *Rab3c*), and regulators of membrane potential and calcium signaling (*Kcnj5*, *Hcn1*, *Cftr*, *Lrrc8*, *Actn2*, *Chrn7a*). Another large cluster of genes (green cluster) contained genes whose upregulation by glucose in control β cells was blunted by *Pcbp2* deficiency. Examination of this cluster revealed genes linked to processes similar to those of the yellow cluster, including cAMP signaling (*Crem*, *Prkar1a*, *Pde8b*), synapse activity (*Bsn*, *Ntrk2*, *Lrrtm2*), membrane potential (*Kcnf1*, *Slc26a3*), intracellular calcium flux (*Casq2*, *Reep5*), and insulin granule formation (*Chgb*). The orange cluster harbored a smaller set of genes that were primarily downregulated in mutant β cells incubated with high glucose (Supplemental Figure 10D). We also found in this module a handful of genes with roles in vesicle transport (*Rin1*, *Fam110c*), ion transport (*Cnih3*), and synapse regulation (*Il1rap*). In contrast to the clusters with downregulated gene patterns, our clustering analysis raised modules of genes that were mainly unaffected by glucose in control β cells but were upregulated irrespective of glucose treatment in mutant β cells (blue cluster; Supplemental Figure 10D) or were uniquely upregulated in mutant β cells with high glucose exposure (black cluster; Supplemental Figure 10E). Interestingly, we found several genes with roles in cell division and replication mapped to these clusters (*Ccnb1ip1*, *Ccne2*, *Ncapd2*, *Supt16*, *Trip13*, *Nsl1*, *Fgf9*). Together, these data implicate PCBP2 in coordinating glucose-dependent and -independent gene regulation to regulate important processes in the β cell.

To examine how PCBP2 impacts the mRNA levels of genes in our data set, we searched the 3′-UTR of the 603 dysregulated transcripts for the presence of PCBP2 binding motifs. PCBP2 can influence steady-state levels of mRNAs through 3′-UTR regulatory control, and the presence of PCBP2 binding sites in this region has linked PCBP2 to posttranscriptional regulation of motif-containing target mRNAs (27). To identify motifs, we used the Multiple EME for Motif Elicitation (MEME) motif-finding algorithm to perform de novo motif analysis to identify enriched motifs in the 3′-UTRs of transcripts altered with *Pcbp2* deficiency following glucose challenge (28). Further, to discriminate between motifs that are general features of 3′-UTRs versus uniquely enriched in the 3′-UTRs of our set, we performed a MEME motif search using a background of an equal number of randomly sampled 3′-UTR sequences from transcripts with no changes in abundance. Remarkably, our motif search returned a single, 18-nucleotide cytosine-rich sequence that was enriched in the 3′-UTRs of the transcripts of 255 genes impacted by loss of PCBP2 (Figure 4D). To investigate the relevance of this motif to PCBP2, we searched this motif against a database of curated RBP motifs and found that the top match to our de novo motif was that of a curated PCBP2 motif (Figure 4E). Altogether, these results point toward direct PCBP2 binding playing a role in regulating the abundance of mRNAs that regulate the functional state of β cells.

### Pcbp2 deficiency in vivo impairs insulin secretion.

**Figure 5:**
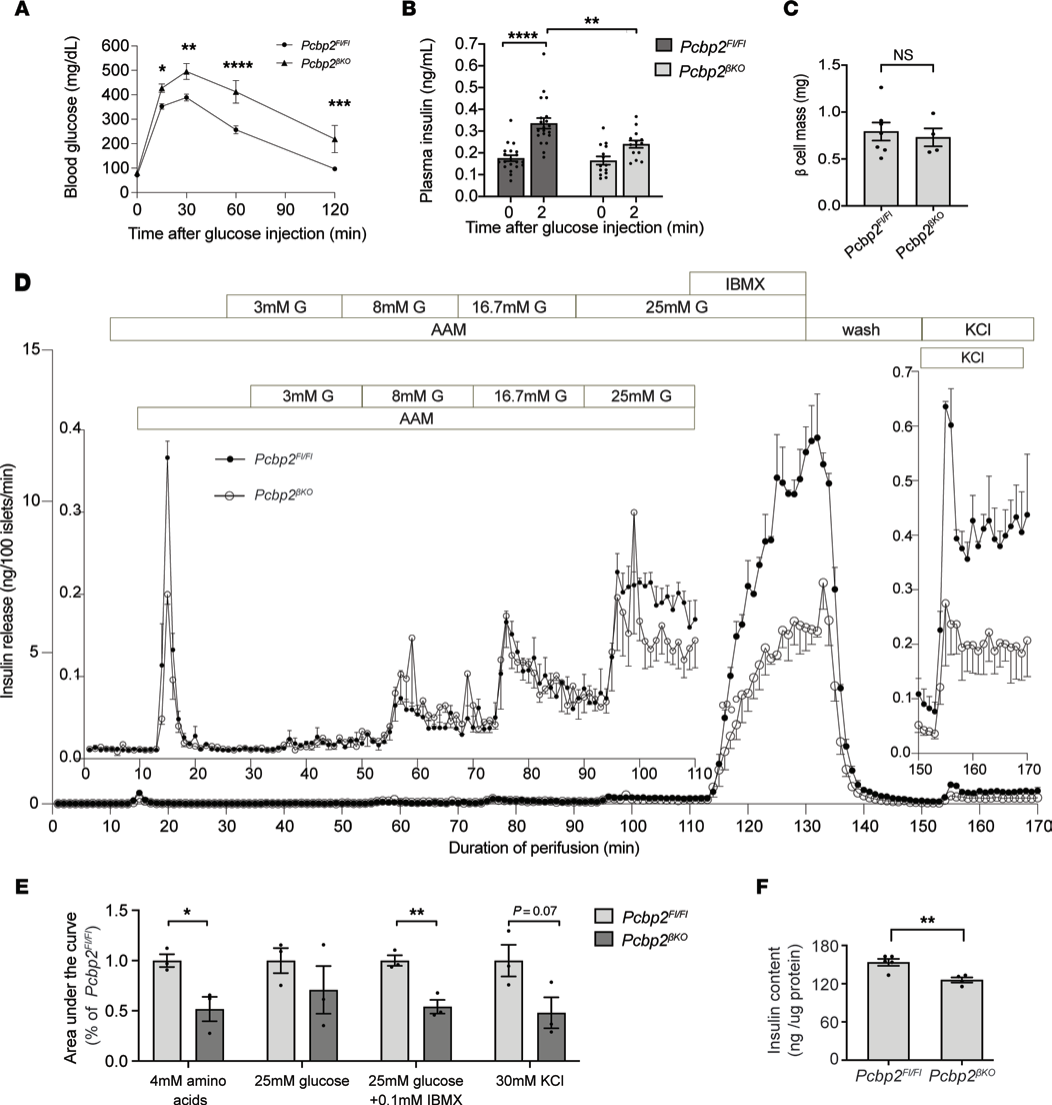
Deleting *Pcbp2* in β cells impairs glucose homeostasis and insulin secretion. (**A**) Intraperitoneal glucose tolerance test on 7- to 8-week-old male mice (*n* = 11 *Pcbp2^fl/fl^*; *n* = 17 *Pcbp2*^βKO^). (**B**) Acute in vivo glucose-stimulated insulin release in 7- to 8-week-old male mice (*n* = 20 *Pcbp2^fl/fl^*; *n* = 14 *Pcbp2*^βKO^). (**C**) Quantified β cell mass from 7- to 8-week-old male mice (*n* = 7 *Pcbp2^fl/fl^*; *n* = 4 *Pcbp2*^βKO^). (**D**) Dynamic insulin secretion of isolated islets from 7- to 9-week-old male mice challenged with 4 mM amino acid mixture (AAM), increasing amounts of glucose (3 to 25 mM), high glucose (25 mM), and 0.1 mM IBMX, and 30 mM KCl (*n* = 3 *Pcbp2^fl/fl^*; *n* = 3 *Pcbp2*^βKO^). (**E**) AUC measurements of total insulin secretion in response to high glucose ± 0.1 mM IBMX and 30 mM KCl (*n* = 3 *Pcbp2^fl/fl^*; *n* = 3 *Pcbp2*^βKO^). (**F**) Insulin content in isolated islets from 7- to 8-week-old male mice (*n* = 5 *Pcbp2^fl/fl^*; *n* = 4 *Pcbp2*^βKO^). *P < 0.05, **P < 0.01, ****P < 0.0001 by 2-way ANOVA with Holm-Šidák post hoc test (**A** and **B**) and by Student’s 2-tailed *t* test (**C**–**F**).

Our RNA sequencing analysis implicated *Pcbp2* in key processes that modulate β cell insulin secretion. Therefore, we sought to determine the functional requirement of *Pcbp2* in the β cell. We generated age- and sex-matched cohorts of *Pcbp2*^βKO^ and littermate controls. *Pcbp2*^βKO^ mice were born at normal Mendelian ratios, survived well into adulthood, and exhibited no differences in body weight gain and ad libitum blood glucose levels (Supplemental Figure 11, A–D). In contrast, *Pcbp2*^βKO^ mice were glucose intolerant when challenged with a glucose bolus, and this defect was more subtle in females, likely because of the protective effects of female sex steroids on glucose homeostasis (29) (Figure 5A and Supplemental Figure 12, A and B). Peripheral tissue insulin sensitivity was normal (Supplemental Figure 13), in agreement with β cell–specific genetic alterations. Further, *Cre* recombinase expression and *Pcbp2 loxP* insertion had no effect on glucose tolerance (Supplemental Figure 14), supporting *Pcbp2* deficiency as the primary driver of the observed glucose intolerance. Consistent with the glucose tolerance defect, *Pcbp2*^βKO^ mice showed reductions in acute glucose-stimulated insulin secretion (Figure 5B). β Cell mass was normal (Figure 5C), indicating a β cell functional defect.

To directly examine the functional defect, we performed ex vivo perifusion assays of *Pcbp2*^βKO^ and *Pcbp2^fl/fl^* islets. Islets were first perifused with an amino acid mixture to stimulate β cell insulin release and to prime these cells for better insulin release from subsequent stimulation (30). Glucose was then added in steps of low (3 mM), intermediate (8 mM), and high (16.7 mM/25 mM) concentrations followed by IBMX and KCl. Amino acid challenge induced a burst in insulin release that was reduced about 50% in *Pcbp2*^βKO^ islets compared with control islets, as determined by area under the curve (AUC) quantification (Figure 5, D and E). In contrast, these mutant islets displayed a normal insulin secretion profile in response to 3 mM, 8 mM, and 16.7 mM glucose challenge but an approximately 30% reduction in response to 25 mM glucose (Figure 5, D and E). Potentiation of insulin secretion by IBMX was about 50% reduced in *Pcbp2*^βKO^ islets, and KCl-stimulated insulin release was similarly blunted (Figure 5, D and E), indicating defects in cAMP-amplified insulin secretion and the readily releasable pool of insulin granules, respectively. Insulin content was slightly reduced, by about 18% (Figure 5F). Thus, *Pcbp2* deficiency impairs insulin secretion to an array of insulin secretagogues and reduces the capacity of glucose-stimulated insulin secretion, agreeing with the findings of our RNA sequencing analysis.

### Intracellular calcium flux and insulin granule ultrastructure and dynamics are impaired in the setting of Pcbp2 deficiency.

The KCl defect in our perifusion data pointed toward *Pcbp2-*deficient β cells harboring defects in release of the readily releasable pool of insulin granules (Figure 5, D and E). Supporting this notion, our transcriptomic analysis demonstrated that *Pcbp2* deficiency downregulated processes that impact readily releasable pool dynamics, including calcium ion transport, vesicle organization, and membrane docking and exocytosis (31). Therefore, we followed up on whether these key processes were perturbed with loss of *Pcbp2*.

**Figure 6:**
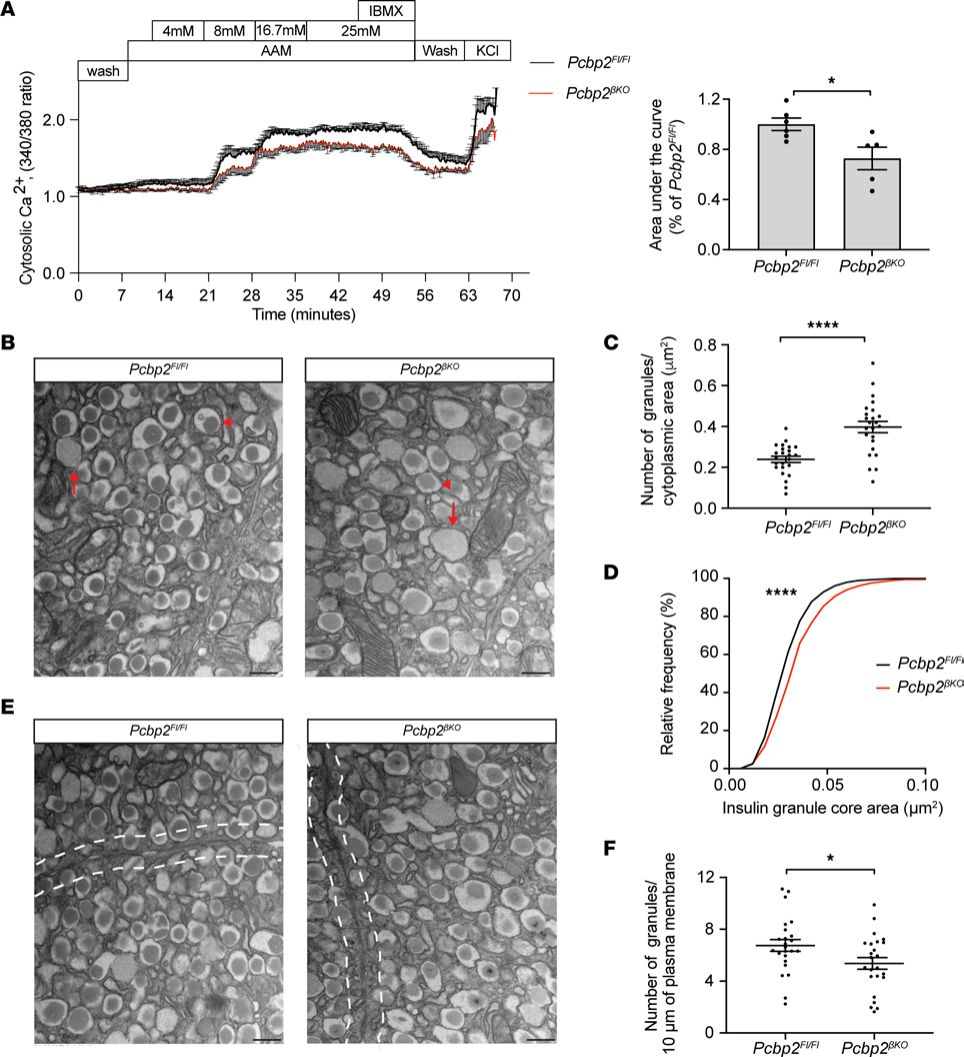
*Pcbp2*-depleted β cells exhibit defective intracellular calcium flux, increased numbers of immature insulin granules, and decreased insulin granule docking. (**A**) Intracellular calcium flux and corresponding AUC measurements from islets isolated from 7- to 9-week-old mice challenged with 4 mM amino acid mixture (AAM), increasing amounts of glucose (3 to 25 mM), high glucose (25 mM), and 0.1 mM IBMX, and 30 mM KCl (*n* = 6 *Pcbp2^fl/fl^*; *n* = 5 *Pcbp2*^βKO^). (**B**) Transmission electron microscopy (TEM) image showing individual insulin granules from control and *Pcbp2-*deficient β cells from 8- to 9-week-old mice. Red arrows indicate immature insulin granules, whereas red arrowheads indicate mature insulin granules (scale bars: 400 nm). (**C** and **D**) Quantification of number of immature insulin granules (**C**) and insulin core areas (**D**) in control and mutant β cells (*n* = 24 images from 3 pooled mice per genotype). (**E**) TEM imaging showing docked insulin granules in control and *Pcbp2-*deficient β cells marked by dashed white lines from islets from 7- to 9-week-old mice (scale bars: 400 nm). (**F**) Quantification of number of docked insulin granules in β cells from each genotype (*n* = 24 images from 3 pooled mice per genotype). *P < 0.05, ****P < 0.0001 by 2-tailed Student’s *t* test (**A**, **C**, and **F**); and ****P < 0.0001 by Kolmogorov-Smirnov test (**D**).

It is well established that all insulin secretagogues converge on modulating either the availability or efficiency of intracellular calcium to elicit insulin secretion, making calcium signaling indispensable for insulin release. Given the critical nature of this cation and the observed downregulation of several regulators of calcium ion homeostasis (*Kcnj5*, *Hcn1*, *Cftr*, *Chrn7a*, *Lrrc8*, *Actn2*) in mutant β cells across basal and glucose-adaptive conditions, we next performed intracellular calcium signaling measurements in *Pcbp2*^βKO^ and control islets. Paralleling the paradigm used in our perifusion experiments, we measured intracellular calcium flux as determined by Fura-2 AM islets exposed to low, intermediate, and high glucose steps followed by IBMX and KCl. Strikingly, *Pcbp2*^βKO^ islets exhibited blunted calcium intracellular flux in response to all administered insulin secretagogues, and AUC quantification showed that overall calcium flux signal was reduced about 30% (Figure 6A). Thus, impaired calcium flux contributes to the defective insulin secretion in *Pcbp2-*deficient β cells.

We next sought to determine whether changes in insulin granule dynamics were altered in *Pcbp2*^βKO^ islets. Transmission electron microscopy was performed on β cells from control and *Pcbp2*^βKO^ islets. Morphometric analysis revealed that *Pcbp2-*deficient β cells harbored increased amounts of enlarged, secretory vesicles exhibiting diffuse, light gray cores as compared with controls (Figure 6B, red arrows).

When quantified, mutant β cells harbored an approximately 1.7-fold increase in the number of these abnormal vesicles (Figure 6C). Interestingly, these secretory vesicles matched the morphometric criteria of immature insulin granules, which exhibit diffuse, light gray and enlarged core areas as opposed to the packed, electron-dense cores of smaller mature insulin granules (32). This parallel observation suggested that *Pcbp2-*deficient β cells harbored increased amounts of immature insulin granules. Supporting this notion, we found an increase in the size distribution of the core areas of the insulin granules harbored in *Pcbp2-*deficient β cells (Figure 6D). Further, we found that *Pcbp2*^βKO^ islets had reduced proinsulin content, a trending increase in the ratio of proinsulin to insulin content, and decreased expression of the proinsulin-converting enzyme *Pcsk1* (Supplemental Figure 15, A–C), features that have been linked to increased pools of immature insulin granules (33, 34). The ultrastructure analysis of mutant β cells further revealed an approximately 20% reduction in the number of insulin granules within 200 nm of the plasma membrane (Figure 6, E and F, dashed white lines), indicating a slight reduction in the docked pool of insulin granules. These results demonstrate that PCBP2 is required to maintain the proper architecture and dynamics of insulin secretory granules in the β cell. Taken together, these data indicate that PCBP2 regulates multiple components of the insulin secretory pathway and that a combination of defects contribute to the impaired insulin release from *Pcbp2-*null β cells.

### PCBP2 regulates insulin vesicle, exocytosis, and insulin gene expression.

**Figure 7:**
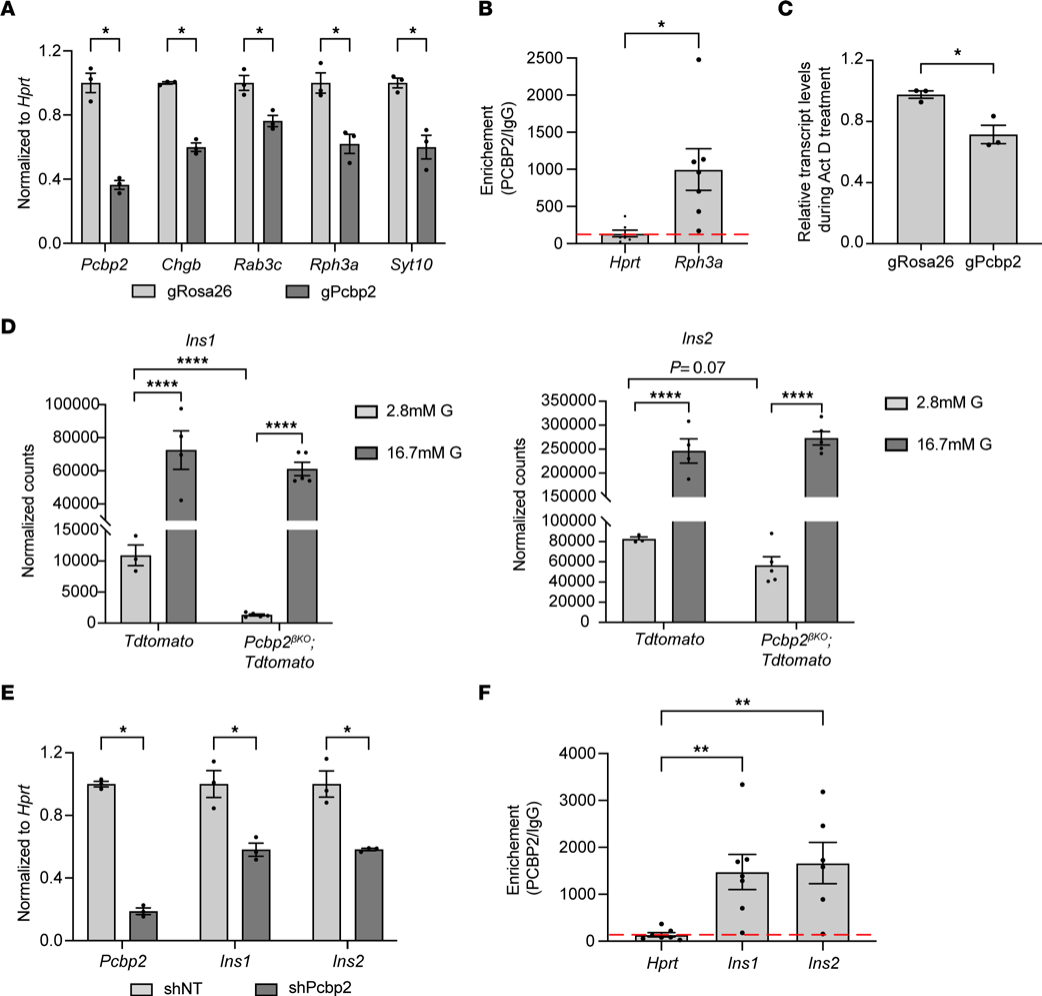
PCBP2 regulates insulin granule, exocytosis, and insulin genes. (**A**) RT-qPCR of select PCBP2-regulated genes in Min6 cells with CRISPR-mediated depletion of PCBP2 (*n* = 3). (**B**) PCBP2 interaction with the mRNA encoding RPH3A compared with that of *Hprt* housekeeping gene (*n* = 7). (**C**) *Rph3a* transcript levels in *Pcbp2-*depleted Min6 cells following transcriptional inhibition with actinomycin D (*n* = 3). (**D**) Normalized expression of *Ins1* and *Ins2* genes in RNA sequencing of FACS-isolated control and *Pcbp2-*deficient β cells during low and high glucose exposure. (**E**) *Ins1* and *Ins2* transcript levels in Min6 cells cultured in 5.5 mM glucose with *Pcbp2* depletion. (**F**) PCBP2 interaction with mRNAs encoding insulin relative to that of housekeeping control *Hprt* in Min6 cells (*n* = 6). Control values in **B** are presented in **F**. *P < 0.05, **P < 0.01, ****P ≤ 0.0001 by Student’s *t* test (**A**–**C** and **E**), edgeR differential analysis (**D**), and 1-way ANOVA with Holm-Šidák post hoc test (**F**).

To investigate mechanisms by which PCBP2 impacts its regulated mRNAs, we first selected a panel of genes with links to insulin granule maturation, trafficking, and membrane docking for further study based on the defects observed in *Pcbp2*^βKO^ islets. Using CRISPR/Cas9 targeting to effectively ablate *Pcbp2* expression in the Min6 β cell model (Supplemental Figure 16), we confirmed that *Pcbp2* deficiency reduced the mRNA abundance of all genes in this panel (Figure 7A). We next selected *Rph3a* as a candidate for further mechanistic investigation since it is key player in docking and fusion of insulin granules and was raised as a putative PCBP2 interactor in our motif analysis (35). RNA immunoprecipitation with α-PCBP2 antisera followed by quantitative reverse transcription PCR (RT-qPCR) revealed a more than 5-fold enrichment of *Rph3a* mRNA compared with that of the housekeeping gene *Hprt* in PCBP2 immunoprecipitates (Figure 7B). We next determined that PCBP2 stabilized *Rph3a* expression by treating control and *Pcbp2-*deficient Min6 cells with the transcriptional inhibitor actinomycin D for 12 hours and then measuring the transcript abundance of *Rph3a* in both groups. This approach revealed that depleting *Pcbp2* reduced *Rph3a* transcript abundance compared with that of control Min6 cells, indicative of decreased mRNA stability (Figure 7C). Thus, PCBP2 regulates *Rph3a* expression by binding and stabilizing the *Rph3a* transcript.

Our findings of reduced insulin and proinsulin content in *Pcbp2-*mutant islets raised the possibility that PCBP2 also directly impacts insulin gene expression. Indeed, *Pcbp2-*null β cells exhibited markedly reduced *Insulin 1* (*Ins1*) and a trending reduction in *Insulin 2* (*Ins2*) levels (Figure 7D). Intriguingly, the influence of PCBP2 on these mRNAs occurred under basal but not during glucose-adaptive conditions. To confirm that PCBP2 impacts *Ins1* and *Ins2* levels under low-glucose conditions, we used Min6 cells cultured long-term in low-glucose-containing medium (5.5 mM) and used a shRNA to effectively deplete *Pcbp2*. In accord with our transcriptomic findings, we observed approximately 50% reduction in *Ins1* and *Ins2* with loss of *Pcbp2* (Figure 7E). Interestingly, *Ins1* and *Ins2* harbor a conserved cytosine-rich sequence in their 3′-UTRs (36) (Supplemental Figure 17), suggesting that the regulation of PCBP2 of the insulin mRNAs involves direct binding. Accordingly, we found more than 10-fold enrichment of *Ins1* and *Ins2* mRNAs in PCBP2 immunoprecipitates (Figure 7F). Taken together, these findings implicate PCBP2 as having direct impact on mRNAs pivotal for insulin production and secretion.

### PCBP2 is induced by glucose in human islets and is downregulated in T2D.

**Figure 8:**
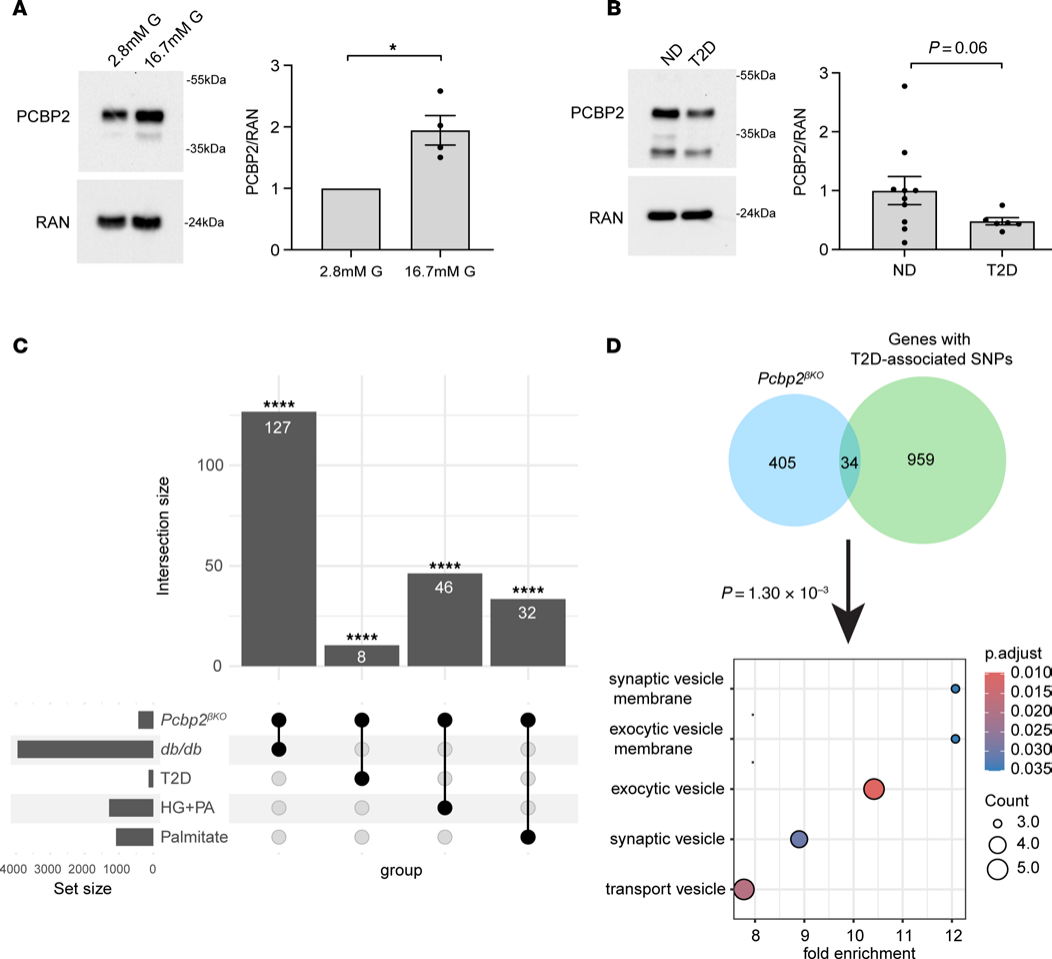
PCBP2 is glucose-induced in human islets and downregulated in T2D. (**A** and **B**) Representative Western blot showing PCBP2 levels in human islets incubated in either 2.8 mM or 16.7 mM glucose for 48 hours (*n* = 4 individual donors) (**A**) and in human islets from control donors (nondiabetic; ND) and donors with T2D (*n* = 10 control; *n* = 6 T2D) (**B**). (**C**) Upset plot showing overlap of genes with decreased transcript levels with *Pcbp2* deficiency during high glucose exposure with those altered in murine and human islets in type 2 diabetic conditions. (**D**) Venn diagram comparing PCBP2-regulated genes with genes with T2D-associated SNPs in the NHGRI-EBI Catalog. *P < 0.05, ****P < 0.0001 by Student’s 2-tailed *t* test (**A**) and hypergeometric tests with FDR-adjusted *P* values (**C**).

Finally, we investigated whether our findings had relevance to human islets and T2D. We first incubated primary, nondiabetic human islets with high glucose levels and found that this stimulation induced a more than 2-fold increase in PCBP2 protein abundance (Figure 8A). This induction mirrored our finding in mouse islets and demonstrates an evolutionary conservation for the glucose regulation of PCBP2. We also observed that PCBP2 was reduced about 50% in human islets from organ donors with T2D as compared with islets from age-matched, nondiabetic donors (Figure 8B and Supplemental Table 3), suggesting that PCBP2 could play a role in the pathophysiology of T2D. Indeed, the genes whose mRNA levels were downregulated with loss of PCBP2 significantly overlapped with genes altered in islets from diabetic *db/db* mice (Figure 8C and Supplemental Figure 18A; *P =* 1.73 × 10^–13^), which include several regulators of membrane potential (*Kcnf1*, *Kcnk10*, *Kcna2*), cAMP signaling (*Grp135*, *Pde7b*, *Cftr*, *Adgrg6*, *Adgrf5*, *Adgrd1*, *Star*, *Igfbp5*, *Stc1*), synapse machinery formation (*Synpr*, *Bsn*, *Myo5c*, *Cttnbp2*), and insulin granule formation (*Chgb*). Further, the PCBP2-regulated genes overlapped those altered in human islets cultured in a type 2 diabetic milieu of palmitate and/or high glucose (Figure 8C and Supplemental Figure 18A; *P =* 3.068 × 10^–3^, *P =* 5.06 × 10^–6^) and in islets from human donors with T2D (Figure 8C and Supplemental Figure 18A; *P =* 3.06 × 10^–3^) (37–39). Among these common genes, we found regulators of cAMP signaling (*Crem*, *Pde8b*) and membrane potential (*Kcna2*, *Kcnj5*). Interestingly, the vesicle docking and membrane fusion regulator *Rph3a*, which we found bound and stabilized by PCBP2 (Figure 7, B and C), was among the common genes altered in islets from human subjects with T2D and islets exposed to palmitate.

Lastly, 34 of the PCBP2-regulated genes contained T2D-associated single-nucleotide polymorphisms (SNPs) or mapped to intergenic SNPs linked to T2D in the curated National Human Genome Research Institute (NHGRI) GWAS Catalog (Figure 8D and Supplemental Figure 18B; *P =* 1.31 × 10^–3^), further underscoring that PCBP2 impacts genes with important roles in shaping glucose homeostasis. Intriguingly, the PCBP2-regulated genes with T2D-associated SNPs were primarily enriched in cellular features relating to synaptic and exocytotic vesicles (*Rph3a*, *Syt10*, *Rab3c*, *Syndig1*, *Cttnbp2*, *Ntrk2*, *Myo5c*; Figure 8D), further bolstering the major influence of PCBP2 on shaping gene expression important for insulin granule ultrastructure and dynamics. Taken together, these findings point to a role for PCBP2 in human islet glucose adaptation and in T2D pathogenesis.

## Discussion

**Figure 9:**
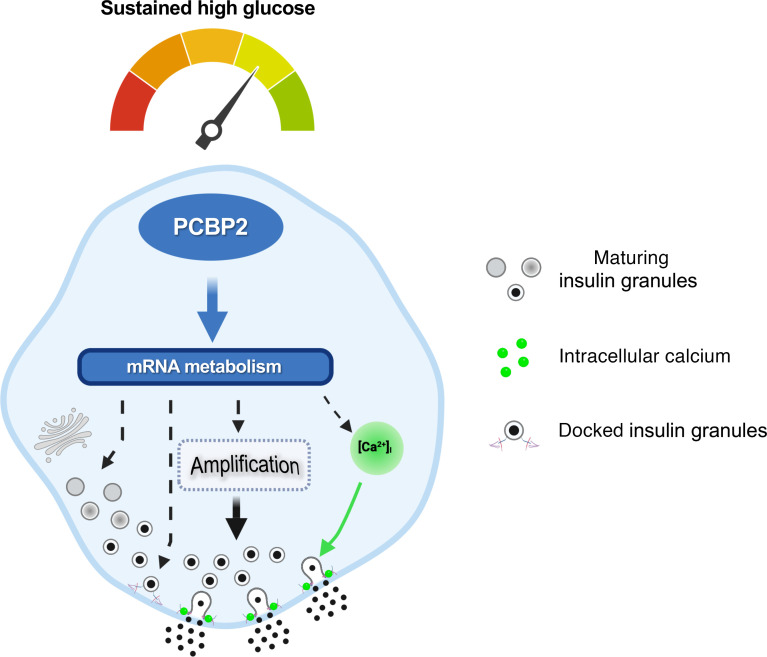
Proposed model of PCBP2 impacting β cell processes required for both basal and glucose-adaptive conditions. PCBP2 governs the metabolism of mRNAs encoding products implicated in shaping multiple components of the insulin secretory pathway. In response to sustained hyperglycemia incubation, PCBP2 is upregulated, which supports glucose-induced transcriptome remodeling, thereby allowing the β cell to augment the insulin secretory response. Model created with BioRender (https://www.biorender.com/).

It is well known that β cells can adjust their insulin secretory capacity in response to changes in metabolic demands, yet the mechanisms that confer the ability of β cells to fine-tune their function remain less resolved. Here, we have discovered that the RBP PCBP2 plays key roles in governing the functional state of β cells. Depleting *Pcbp2* in β cells impaired insulin secretion resulting from defects in calcium flux, insulin production, insulin granule ultrastructure and exocytosis, and the amplification pathway of insulin secretion. Transcriptomic analysis supported these findings and, coupled with motif enrichment analysis, mRNA interaction, and stability studies, revealed that PCBP2 impacts these β cell features in part through direct binding and mRNA stabilization. PCBP2 was elevated in both human and mouse islets challenged with sustained high-glucose conditions, and depleting PCBP2 in the β cell impaired glucose adaptation of the amplification pathway of insulin secretion. By interrogating the β cell transcriptome of control and *Pcbp2-*deficient β cells under basal and glucose-adaptive conditions, we further demonstrated that PCBP2 exhibits a complex regulatory pattern of control on the mRNAs abundance of genes required for both the preservation and functional enhancement of β cells. Together, these results suggest a model whereby PCBP2 posttranscriptionally regulates gene programs necessary to modulate the functional capacity of β cells during acute as well as sustained high-glucose challenge (Figure 9).

The broader implications of our findings suggest a role for PCBP2 in β cell compensation, which goes awry during the progression of T2D. In this adaptation phase, β cells undergo functional changes that augment the sensitivity and capacity for stimulus-coupled insulin secretion in response to excess nutrient availability and/or insulin resistance (40). Here, we observe that PCBP2 is required for amplifying insulin secretion during increased metabolic fuel availability and that *Pcbp2* deficiency impacts the levels of mRNAs that encode proteins involved in boosting stimulus-coupled insulin secretion, including calcium flux, vesicle production and docking, and exocytosis. Indeed, these features have also been implicated in compensating β cells in vivo (41–43). These parallel observations point toward PCBP2 playing a protective role against β cell failure. Indeed, the conserved glucose induction of PCBP2 in nondiabetic human islets and dysregulation of this factor in islets from subjects with T2D further support this notion. It is also interesting to note that PCBP2 decreases in islets from organ donors with T2D despite the hyperglycemia commonly experienced by these individuals. These discordant observations could be due to the chronicity of high glucose experienced in individuals with T2D, which is well established to exert deleterious effects on islet physiology and gene expression. It is also possible that a defect in the glucose induction of PCBP2 is integral to T2D pathophysiology. Thus, it will be worthwhile to decipher the causative factor(s) leading to reduced PCBP2 in T2D islets and whether manipulating PCBP2 and/or activity can preserve the adaptive β cell response against the excess nutrient milieu in vivo observed in T2D.

We found that the stability of PCBP2 protein increases in primary islets challenged with sustained high glucose levels (Figure 1, A–D). The PCBPs are known to be sensitive to changes in extracellular and intracellular cues, which can alter their influence on mRNA metabolism (21, 44). These regulatory changes have been attributed to complex patterns of posttranslational modifications that can drastically alter PCBP levels, activity on target mRNAs, and cellular compartmentalization (21, 45). Therefore, it is plausible that glucose imparts posttranslational modification(s) on PCBP2 to promote increased stability of this protein. Further, these glucose alterations may be shared among other PCBP members. The study that raised PCBP2 as a putative glucose-regulated protein in human islets also identified PCBP3, a less well-studied sister isoform of the PCBP family, as a potential glucose-regulated protein (23). Since PCBP2 regulates the levels of mRNAs broadly required for β cell function, it will be worthwhile to further decipher the mechanisms by which glucose modulates the stability of this factor and whether this regulation extends to other members of the PCBP family.

Here, we implicate PCBP2 as a direct regulator of *Ins1* and *Ins2* expression under basal but not glucose adaptation conditions (Figure 7D). It is currently unclear how PCBP2 impacts the insulin mRNAs and why this impact is inversely related to glucose concentration. It is possible that other RBPs exhibit a dominant influence and/or compensate in the absence of PCBP2 to support insulin expression during glucose-adaptive conditions. Supporting this notion, closely related PCBP2 family members PCBP1 and hnRNPK interact with *Ins1* mRNA (46). Additionally, the impact of PCBP2 on steady-state *Ins1* and *Ins2* mRNA levels and PCBP2 interactions with these mRNAs raises the notion that this factor exerts a stabilizing effect. However, our attempts to assess this possibility with transcriptional inhibition were hindered by the long-lived and stable nature of the insulin mRNAs, as previously noted (36).

One intriguing finding from our perifusion studies was that *Pcbp2-*null β cells exhibited defective insulin secretion when acutely challenged with high but not lower concentrations of glucose (Figure 5D). This dosage-dependent defect was unexpected given that these mutant β cells exhibit defects in intracellular calcium flux and insulin granule docking, which are integral to insulin secretion (Figure 6, A and E). These discordant observations could be due to the metabolic amplifying properties of glucose, which increase the efficiency of insulin granule docking at the plasma membrane and calcium-mediated exocytosis of insulin granules (47). Interestingly, the amplification phenomenon is maximally activated by 20 mM to 30 mM glucose challenges in isolated mouse islets (48). Therefore, it is plausible that under lower glucose concentrations, *Pcbp2-*deficient β cells can mount a normal insulin secretory response, but under higher glucose concentrations when accelerated insulin secretion depends on maximal activation of the metabolic amplification pathway, the insulin secretory capacity of *Pcbp2-*deficient β cells is limited.

PCBP2 exhibits a remarkable range of functions and thus likely plays multiple roles in the β cell. This protein coordinates several layers of gene regulation, such as mRNA transcription, splicing, stability, and translation (71). Further, this factor can achieve its diversity of regulation through direct and indirect effects on its target mRNAs (27). Supporting this notion, our studies reveal that PCBP2 has direct impact on mRNAs harboring a PCBP2 binding site while also affecting the levels of mRNAs lacking this sequence element. The indirect effect could reflect PCBP2 affecting the expression of other RBPs, which could also affect mRNA abundance, a common mechanism employed by RBPs to fine-tune gene expression (49). Indeed, several other RBPs were altered in our RNA sequencing data set (*Rbms3*, *Rbmx2*, *Rbm3*, *Ddx3*). Given the versatile nature of PCBP2, it will be worthwhile to determine how and to what extent this RBP more broadly impacts mRNA metabolism in the β cell.

In conclusion, our study addresses a critical gap in the understanding of how β cells maintain and adapt their functional status in response to hyperglycemia. We identify the RBP PCBP2 as a glucose-induced protein in β cells, and we find that PCBP2 governs the abundance of mRNAs encoding products that impact core processes of the insulin secretory pathway required for acute and adaptive glucose-stimulated insulin secretion. Impairment of β cell function is a key determinant in the development of diabetes, and the identification of mechanisms that modulate and sustain the β cell against detrimental effects of hyperglycemia is of intense therapeutic interest. Given that our findings implicate PCBP2 in shaping key β cell gene expression and β cell function under broad conditions of glucose stimulation, targeting of this protein presents as an appealing potential therapeutic to combat T2D.

## Methods

### Sex as a biological variable.

Our study examined male and female animals, and sex-dimorphic effects are reported.

### Animal models.

Mice were maintained on a 12-hour light/12-hour dark cycle with ad libitum access to water and standard laboratory chow in the animal care facility at the University of Pennsylvania. All experiments were performed on male mice unless otherwise indicated. Mice carrying conditional *Pcbp2* alleles (*Pcbp2^fl/fl^*) were crossed with RIP-Cre transgenic mice to generate mice with β cell–specific deletion of *Pcbp2* (*Pcbp2*^βKO^). *Pcbp2^fl/fl^* and RIP-Cre mice were provided by Stephen Liebhaber (University of Pennsylvania, Philadelphia, PA, USA) and Pedro Herrera (University of Geneva, Geneva, Switzerland) respectively (25, 50). For FACS, *Pcbp2*^βKO^ were mated to transgenic mice carrying the Cre-inducible tdTomato reporter alleles (*Pcbp2*^βKO^ *tdTomato*). *tdTomato* mice were acquired from The Jackson Laboratory (51). All mice were maintained on a C57BL/6 background.

### Body weight, blood glucose, and circulating insulin measurements.

Mice were fasted for 16 hours and injected intraperitoneally with a 2 g/kg glucose bolus for intraperitoneal glucose tolerance measurements. Blood glucose was measured with a handheld glucometer and measured at 0, 15, 30, 45, 60, and 120 minutes after injection. For insulin tolerance measurements, mice were fasted 6 hours and injected intraperitoneally with a 0.75 U/kg insulin solution (Novolin, 0169-1834-11), and blood glucose was measured at 0, 15, and 30 minutes after injection. Ad libitum blood glucose and body weight measurements were taken weekly between 5 pm and 6 pm. For glucose-stimulated insulin secretion, mice were fasted for 16 hours, and blood was collected at 0 and 2 minutes after an intraperitoneal glucose bolus injection (3 g/kg) using EDTA-coated tubes (Sarstedt, 16.444.100). Blood was centrifuged for serum separation, and insulin was measured by insulin ELISA (Crystal Chem, 90082).

### Immunohistochemistry and β cell mass measurements.

After euthanasia, pancreata were harvested and fixed in 4% paraformaldehyde for 24 hours at 4°C and were then embedded in paraffin for sectioning. Antigen retrieval was performed using sodium citrate, pH 6, with 0.05% Tween 20 in a pressure cooker. For isolated islets, islets were fixed in 4% paraformaldehyde for 30 minutes at room temperature, resuspended in agarose gel, and embedded in paraffin for sectioning as previously described (52). Primary antisera were applied overnight at 4°C, and secondary fluorophore-conjugated antisera were applied for 2 hours at room temperature. Immunofluorescence was visualized and captured using either a Nikon Eclipse E600 microscope equipped with a QImaging Q/Click digital camera and MetaMorph software or a Keyence BZ-X810 All-in-One fluorescence microscope with Keyence analysis software. See Supplemental Table 4 for antiserum details and dilutions.

For β cell mass calculations, 3 approximately 200-μm-spaced maximal-footprint tissue sections were analyzed per 7- to 8-week-old animal. Sections were scanned using an Aperio slide scanner and quantified using Imagescope software (Leica Biosystems). β Cell mass was determined by determination of the fraction of total insulin-positive area/total footprint area and multiplication of this fraction by the pancreas wet weight measured at harvest.

### Cell culture and Pcbp2 depletion.

Min6 mouse insulinoma cells (passage 30–35) were cultured in high-glucose (25 mM) DMEM (Gibco, 11965084) or low-glucose (5.5 mM) DMEM (Gibco, 11966025), both of which were supplemented with 110 mM sodium pyruvate, 10% FBS, 1% penicillin-streptomycin, and 50 μM β-mercaptoethanol. HEK293T cells were cultured in DMEM containing high glucose. For transcript stability experiments, Min6 cells were treated with actinomycin D (Sigma-Aldrich, A9415-5MG) at 10 μg/mL for 12 hours. An optimal shRNA sequence targeting *Pcbp2* was selected from a pool of several tested shRNA sequences generated using the shERWOOD algorithm (53) (non-targeting, GCGCGATAGCGCTAATAATTT; *Pcbp2*, CACTTCTCATGAACTCACCAT). Rat insulin II promoter, mCherry, and shRNA sequences in an UltramiR miR-30 scaffold were cloned into pLenti CMV (54). For CRISPR experiments, an optimal guide RNA targeting *Pcbp2* was designed with the MIT CRISPR design tool (*Rosa26*, AAGATGGGCGGGAGTCTTCT; *Pcbp2*, ATCTGTTAAGAAGATGCGCG). Guide RNAs were cloned into lentiCRISPR v2, as previously described (55). Plasmids were transfected into 293T cells along with lentiviral packaging plasmids, psPax2 and pMD2.G, for 8 hours in OptiMEM with Lipofectamine 2000 (Invitrogen, 11668019). Medium was then changed to high-glucose DMEM and harvested 2 and 3 days after transfection. Lentivirus was pelleted via ultracentrifugation at 43,262 *g* for 1.5 hours at 4°C and resuspended in MIN6 medium. Lentiviral medium was supplemented with Polybrene (Santa Cruz Biotechnology, sc-134220) at 8 μg/mL, and MIN6 cells were transduced for 6 hours.

### Human and mouse pancreatic isolation and culture.

Human islets from diabetic and nondiabetic donors were obtained through the NIH-supported Human Pancreas Analysis Program and Integrated Islet Distribution Program. Islets were isolated at NIH-approved centers with informed consent and IRB approval. See Supplemental Table 3 for donor islet characteristics.

For murine islet isolation, 0.5 mg/mL collagenase P (Roche, 11213873001) diluted in 1× HBBS with 0.02% BSA was injected into the common bile duct to perfuse the whole pancreas. Perfused pancreata were incubated at 37°C for 16 minutes with gentle shaking. Islets were separated from digested acinar tissue using density gradient centrifugation with Ficoll-Paque (GE Healthcare, 45-001-751) and hand-picked clean 3–4 times. Islets were harvested for either RNA/protein or insulin/proinsulin content (Crystal Chem, 90082; Mercodia, 10-1232-01). For functional assays and sequencing experiments, islets were first recovered for 3 days in 11 mM glucose–RPMI 1640 supplemented with 10% FBS, 2 mM l-glutamine, 23.8 mM sodium bicarbonate, 1% antibiotic antimycotic (Thermo Fisher Scientific, 15240096) with pH adjusted to 7.3.

### Islet transductions.

Islets were isolated from 8- to 10-week-old wild-type male mice, hand-picked clean of acinar, and allowed to recover for 2 hours in RPMI 1640 medium. Islet transductions were then performed as previously described (56). Groups of 200 islets were incubated with 250 mg/mL of trypsin for 2 minutes and gently mixed. Islets were then placed in 5 mL polystyrene tubes (Falcon, catalog 14-959-A) with serum-free RPMI 1640. Lentivirus containing the shRNA vector targeting *Pcbp2* or non-targeting was titered by ELISA (Takara, 631231) and applied at a multiplicity of infection of 20, as previously performed (24). Transduced islets were recovered for 7 days in RPMI 1640 before changing of glucose culture conditions followed by hormone secretion measurements.

### Islet static incubation and perifusions.

For static incubations, groups of 30 islets from 13- to 17-week-old wild-type male mice were washed 3 times with Krebs-Ringer bicarbonate buffer (KRBB) supplemented with 2.8 mM glucose and 0.1% BSA, then equilibrated for 1 hour in KRBB for 1 hour at 37°C with 5% CO_2_. Islets were then incubated in 25 mM glucose ± 0.1 mM 3-isobutyl-1-methylxanthine (IBMX), washed in KRBB, and then incubated in 30 mM KCl. Islets were incubated for 45 minutes in each condition, and culture medium was collected for measurement of ELISA for insulin (Crystal Chem, 90082) or glucagon (Crystal Chem, 81518). At the end of the experiment, islets were harvested for measurement of insulin and glucagon content, also by ELISA.

Perifusion studies were performed using 750 islets from 7- to 9-week-old male *Pcbp2^fl/fl^ and Pcbp2*^βKO^ mice. Islets were pre-perifused with substrate-free medium, and then an amino acid mixture (totaling 4 mM concentration) was added as previously described (57). Next, islets were stimulated with increasing amounts of glucose in a stepwise manner (3 mM to 25 mM glucose). During the 25 mM glucose step, 0.1 mM IBMX was added to further potentiate insulin secretion. Lastly, islets were briefly washed with substrate-free medium to remove all stimuli, and 30 mM KCl was added. Insulin in perifused aliquots was measured by radioimmunoassay at the Radioimmunoassay and Biomarkers Core of the Penn Diabetes Research Center.

### Calcium imaging.

Islets from 7- to 8-week-old *Pcbp2^fl/fl^* and *Pcbp2*^βKO^ mice were loaded with Fura-2 acetoxymethylester (AM) for 40 minutes at 37°C in 3 mL KRBB supplemented with 5 mM glucose and 16.7 μM Fura-2 AM. After incubation, the islets were transferred to a perifusion chamber and placed on a homeothermic platform on an inverted Zeiss microscope (Axio Observer.Z1). Islets were perifused with KRBB at 37°C at a flow rate of 1 mL/min. After recording in the absence of substrate, amino acids (4 mM) alone and then in combination with increasing concentrations of glucose (4 mM, 8 mM, 16.7 mM, and 25 mM) were applied. IBMX was next applied in the presence of amino acids (4 mM) and glucose (25 mM). All substrates were washed away, and KCl (30 mM) was applied. Intracellular Ca^2+^ was measured by dual-wavelength fluorescence microscopy using the Zeiss AxioVision system, as previously described (58).

### Electron microscopic insulin granule analysis.

Islets isolated from three 8- to 9-week-old male *Pcbp2^fl/fl^* and *Pcbp2*^βKO^ mice were pooled into their respective genotype and hand-picked clean 3–4 times in 1× HBSS supplemented with 2.8 mM glucose and 0.2% BSA. Islets were fixed in EM fixative (2% paraformaldehyde, 2.5% glutaraldehyde [Electron Microscopy Sciences, 16220], 3 μM CaCl_2_, and 0.1 M sodium cacodylate [Electron Microscopy Sciences, 12300], pH 7.4) for 1 hour at room temperature and then overnight at 4°C. After subsequent buffer washes, the samples were postfixed in 2.0% osmium tetroxide with 1.5% K_3_[Fe(CN)_6_] for 1 hour at room temperature and rinsed in dH_2_O before en bloc staining with 2% uranyl acetate. After dehydration through a graded ethanol series, the tissue was infiltrated and embedded in EMbed-812 (Electron Microscopy Sciences). Thin sections were stained with uranyl acetate and Sato’s lead (Electron Microscopy Sciences) and examined with a JEOL 1010 electron microscope fitted with a Hamamatsu digital camera and AMT Advantage NanoSprint 500 software.

For quantification of transmission electron microscopy images, individual β cells in *Pcbp2^fl/fl^* and *Pcbp2*^βKO^ mice were scored. Immature insulin granules were identified as previously defined (59). The number of immature insulin granules was quantified and divided by the total cytoplasmic area of measurement. In parallel, insulin granule core areas were scored across individual β cells. Docked insulin granules were determined by measurement of the number of insulin granules with a core center distance of ≤200 nm to the plasma membrane and normalization of this value to the length of plasma membrane examined (60).

### Western blot analysis.

Islets were collected and lysed in RIPA buffer (150 mM NaCl, 50 mM Tris-HCl, 1% NP-40, 0.5% sodium deoxycholate, 0.1% SDS) supplemented with protease and phosphatase inhibitors (MilliporeSigma, 539134 and 524625-1SET). For cycloheximide stability assays, 300 μg/mL of cycloheximide (MilliporeSigma, C4859) was administered for the desired times prior to harvest. Protein concentrations were determined by bicinchoninic acid assay. Between 3 and 10 μg of protein lysates were mixed with Laemmli buffer and boiled for 10 minutes at 70°C. Twelve percent SDS-PAGE gels were used for separation. After protein transfer, nitrocellulose membranes were blocked with PBS-T with 5% milk and incubated overnight at 4°C with shaking. Membranes were exposed to secondary antisera for 2 hours at room temperature, and blots were visualized with Luminate Crescendo HRP substrate (EMD Millipore, WBLUR0100) using the Bio-Rad ChemiDoc Touch Imaging System. Blots were quantified using Bio-Rad Image Lab software. PCBP2 *t*_1/2_ measurements were performed as previously described (61). PCBP2 levels were measured 0, 8, 24, 32, 48, 56, and 60 hours after cycloheximide treatment. Relative intensity values were then fit to a first-order exponential function to estimate the decay constant that was subsequently used to measure PCBP2 half-life. Antisera and dilutions are documented in Supplemental Table 4.

### Cell sorting and RNA sequencing analysis.

After incubation in either 2.8 mM or 16.7 mM glucose–RPMI 1640 medium, *tdTomato* and *Pcbp2*^βKO^ *tdTomato* islets were dispersed into single cells using TrypLE express (Gibco, 12604013). Dispersed islet cells were washed with 1× dPBS and resuspended in FACS buffer (25 mM HEPES, 1% BSA, 1 mM EDTA), pH 7.3, and filtered into 5 mL FACS tubes to further induce a single-cell suspension. DAPI was added (3 μM), and a Beckman Coulter MoFlo Astrios cell sorter was used to enrich for single tdTomato^+^ and DAPI^–^ cells directly into TRIzol LS (Invitrogen, 12604013). A minimum of 14,000 cells were sorted for each sample. RNA was isolated, RNA sequencing libraries were prepared using an Illumina Ultra Low Input RNA kit, and libraries were sequenced on a HiSeq 2500 system using a 150-nt paired-end read protocol (GeneWiz).

Reads were aligned to GRCm39 (mouse) reference transcriptome using Kallisto (62). Transcripts-per-million read counts were collapsed to gene-level counts using tximport R package (63), and genes exhibiting low expression were removed. Differential gene expression analysis was performed with the edgeR package (64) using a design matrix with a factor to control for batch effect (~batch + treatment). Genes with transcript levels undergoing a fold change greater than 1.5 and false discovery rate (FDR) less than 0.05 were considered significant. Heatmaps were created with the heatmap.2 (65) and ComplexHeatmap (66) R packages using *z* score–normalized read counts across samples for each gene. Gene set enrichment analysis (GSEA) was performed with gene sets from the mouse Molecular Signatures Database (MSigDB) (26). Gene ranks were based on the sign(log_2_FC) × –log_10_(*P* value) ranking method, as previously described (67, 68). Gene sets were compiled to assess enrichment of gene ontology (biological processes) and clustering of enriched terms using the ClusterProfiler package in R (69). Gene sets with an FDR less than 0.05 were considered significant.

Genic and intergenic SNPs associated with T2D and their linked genes were extracted from the curated NHGRI-EBI Catalog database for subsequent analysis (70). Hypergeometric tests with post hoc multiple testing corrections were used to determine the significance of overlap between RNA sequencing data sets.

### RNA isolation.

Min6 cells were washed 3 times with cold 1× PBS and then placed in TRIzol, and RNA was extracted according to the manufacturer’s instructions (Invitrogen). High Capacity cDNA Reverse Transcription Kit (Applied Biosystems, 4374966) with random hexamers was used to reverse-transcribe RNA into cDNA. Transcript abundance was measured by real-time qPCR on a QuantStudio 5 (Applied Biosystems), and fold changes were calculated using the ΔΔCt method, with *Hprt* as the normalization control. For mRNA stability measurements, relative transcript abundance was measured during 12 hours of actinomycin D treatment (10 μg/mL). Primer sequences used for RT-qPCR are listed in Supplemental Table 5.

### RNA immunoprecipitation.

RNA immunoprecipitation was performed as previously described (71). Mouse α-PCBP2 (Abnova, H00005094-M07) or IgG antibodies (Invitrogen, 10-400-C) were conjugated to Protein G Dynabeads (Invitrogen, catalog 10001D) in a buffer containing 50 mM Tris-HCl (pH 7.4), 150 mM NaCl, 1 mM MgCl_2_, and 0.05% NP-40. Min6 cell lysate was incubated with antibody-bound Dynabeads overnight with rotation at 4°C. The following day, immunoprecipitates were washed with polysome lysis buffer (100 mM KCl, 5 mM MgCl_2_, 10 mM HEPES-NaOH [pH 7], 0.5% NP-40, 1 mM DTT supplemented with protease, phosphatase, and RNase inhibitors), and RNA was eluted from beads in TRIzol, extracted per the manufacturer’s directions, and reverse-transcribed (Applied Biosystems, 4374966). Transcript enrichment was calculated by normalization of immunoprecipitated RNA to total RNA relative to that of IgG control. See Supplemental Table 5 for primer sequences.

### Motif analysis.

Motif discovery was performed using MEME motif-finding software (28). 3′-UTR sequences were downloaded from the Genome Reference Consortium Mouse Build 39. For background control, a position-specific prior (PSP) was generated using the 3′-UTRs of interest and an equal number of background 3′-UTR sequences from transcripts with detectable expression but not meeting the criterion for differential expression. MEME was then run using the PSP to search for the top 10 motifs using the DNA alphabet and a Markov order of 1. Identified motifs were then searched against the CISBP-RNA single-species RNA (DNA-encoded) database of known RBP motifs.

### Statistics.

Unpaired 2-tailed Student’s *t* test was used where comparison between 2 samples was required. For comparisons with more than 2 groups, 1-way ANOVA with multiple post hoc correction was performed. Two-way repeated-measures ANOVAs with post hoc pairwise comparisons were performed where comparisons between more than 2 groups and repeated measures were being examined. Overlap between gene sets was determined using hypergeometric tests with an FDR less than 0.05 considered significant. Data are presented as mean ± SEM unless otherwise noted. Statistical analyses were performed using GraphPad Prism 9. Outliers were identified by Grubbs’s method and removed. *P* values less than 0.05 were considered statistically significant.

### Study approval.

Animal studies followed institutional guidelines and were approved by the University of Pennsylvania Institutional Animal Care and Use Committee.

### Data availability.

Data are available in the Supporting Data Values file. RNA sequencing data that support the findings of this study were deposited in the NCBI’s Gene Expression Omnibus (GEO) database under accession code GSE263908.

## Author contributions

MWH conceived of, designed, and performed a majority of the experiments and bioinformatics analysis, interpreted results, and drafted the manuscript. AVS, AVR, MJV, MK, SS, NMD, and XY designed and performed experiments and reviewed the manuscript. KLW provided guidance for bioinformatics analyses and reviewed the manuscript. ALG, LRG, and LS provided resources and reviewed the manuscript. DAS conceived of and designed the studies, interpreted the results, and edited the manuscript.

## Supplementary Material

Supplemental data

Unedited blot and gel images

Supplemental table 1

Supplemental table 2

Supplemental table 3

Supporting data values

## Version 1 06/17/2024

Electronic publication
