# Supplementary material for: RNA-binding protein PCBP2 regulates pancreatic β cell function and adaptation to glucose
Source: J Clin Invest. 2024 Jun 17;134(12):e172436. doi: 10.1172/JCI172436 (PMC11178539; doi:10.1172/JCI172436)
Supplement: Supplemental data [file jci-134-172436-s010.pdf]

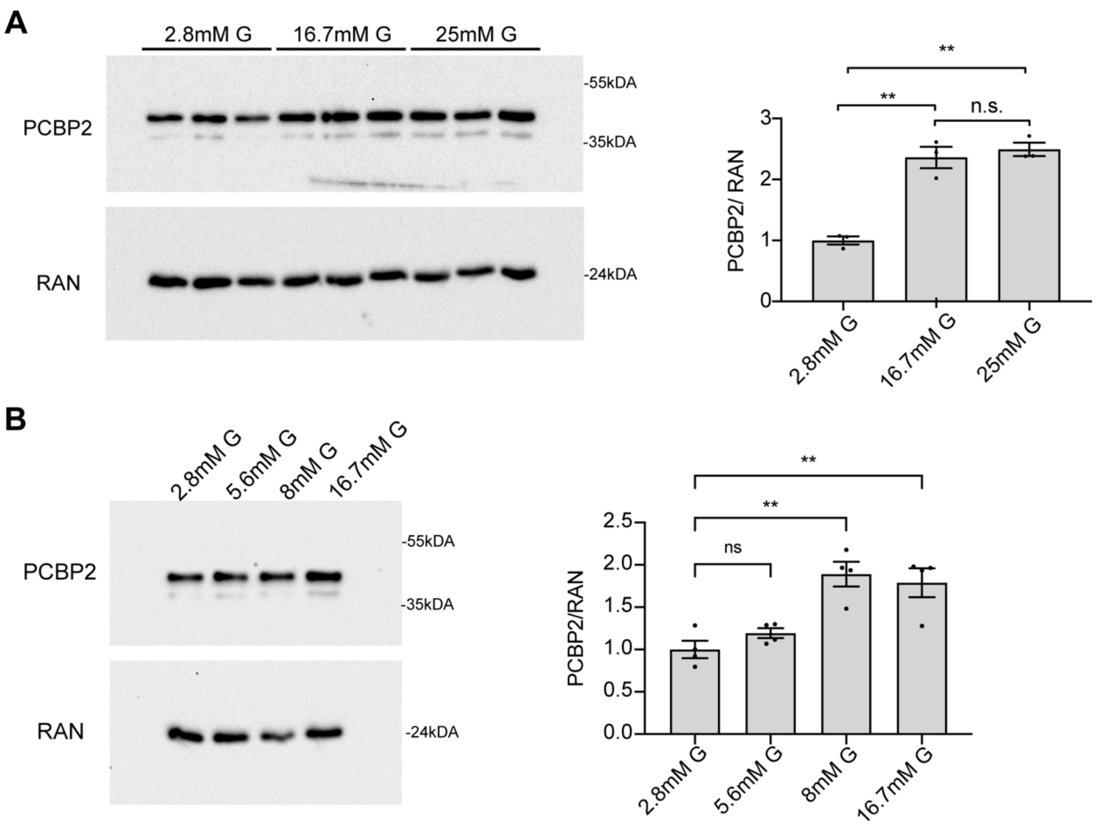

Supplemental Figure 1. PCBP2 increases over a physiologically relevant range of glucose concentrations.

(**A**) Western blot and quantification comparing PCBP2 levels in islets from 12 week old male mice incubated in 2.8mM, 16.7mM, or 25mM glucose for 48 hours (n=3). (**B**) Representative western blot comparing the induction of PCBP2 in islets from 10 week old male mice exposed to increasing steps of 2.8, 5.6, 8, or 16.7mM glucose for 72 hours and quantification of 4 biological replicates. **P-value<0.01 by one-way ANOVA with Holm-Sidak post-hoc correction (**A-B**).

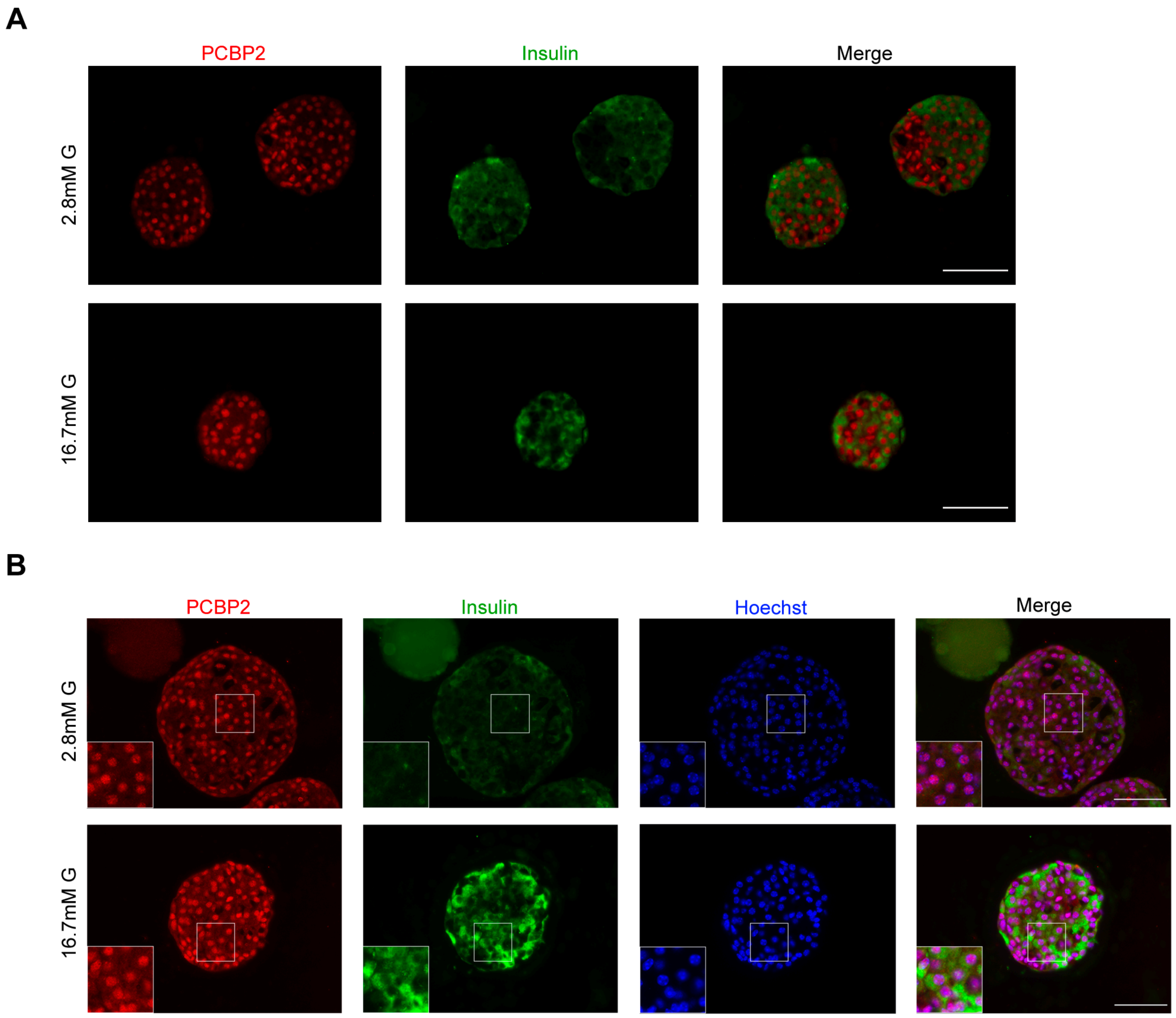

Supplemental Figure 2. PCBP2 abundance appears glucose responsive specifically in β cells and does not undergo overt changes in cellular localization during hyperglycemia. Representative islets from 8-12 week old wildtype male mice incubated with 2.8mM or 16.7mM glucose for 72 hours and stained for (**A**) PCBP2 and Insulin and (**B**) PCBP2, Insulin, and Hoechst (scale bar, 50 μm).

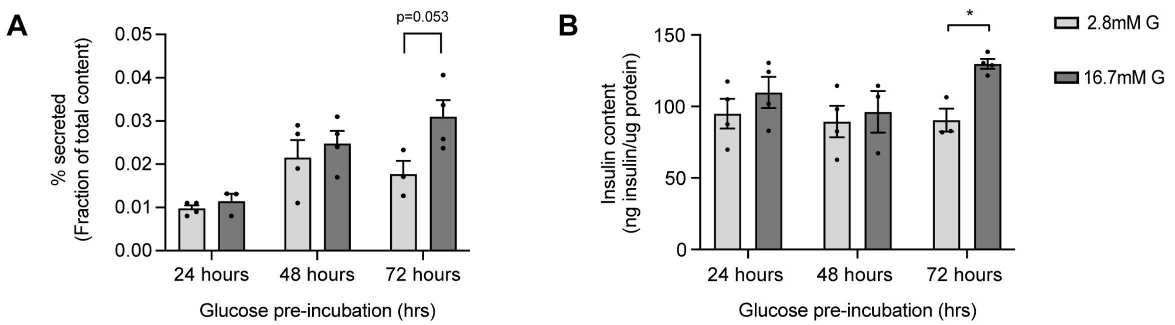

Supplemental Figure 3. Sustained high glucose exposure increases basal insulin secretion and insulin content.
(**A**) Static insulin secretion in response to 2.8mM glucose and (**B**) insulin content profiles of islets from wild-type 13-17 week old male mice preincubated with 2.8mM or 16.7mM glucose for the indicated time periods (n=3-4). *P-value<0.05 by student's two-tailed t-test (**A-B**).

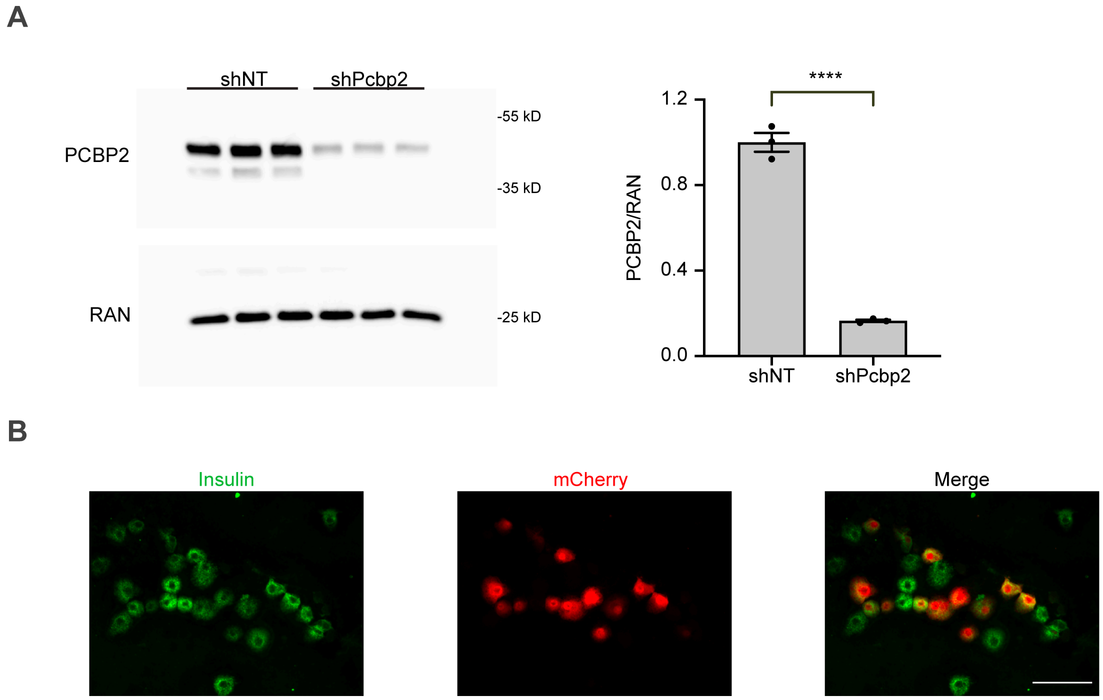

Supplemental Figure 4. Lentiviral shRNA vector effectively depletes PCBP2 in β cells.
(**A**) Western blot showing shRNA-mediated depletion of PCBP2 in Min6 cells (n=3).
(**B**) Co-immunofluorescence staining for Insulin and mCherry on dispersed mouse islet cells following lentiviral delivery of shRNA vector targeting PCBP2 to intact islets (scale bar, 50 μm).
***P-value<0.001 by student's t-test.

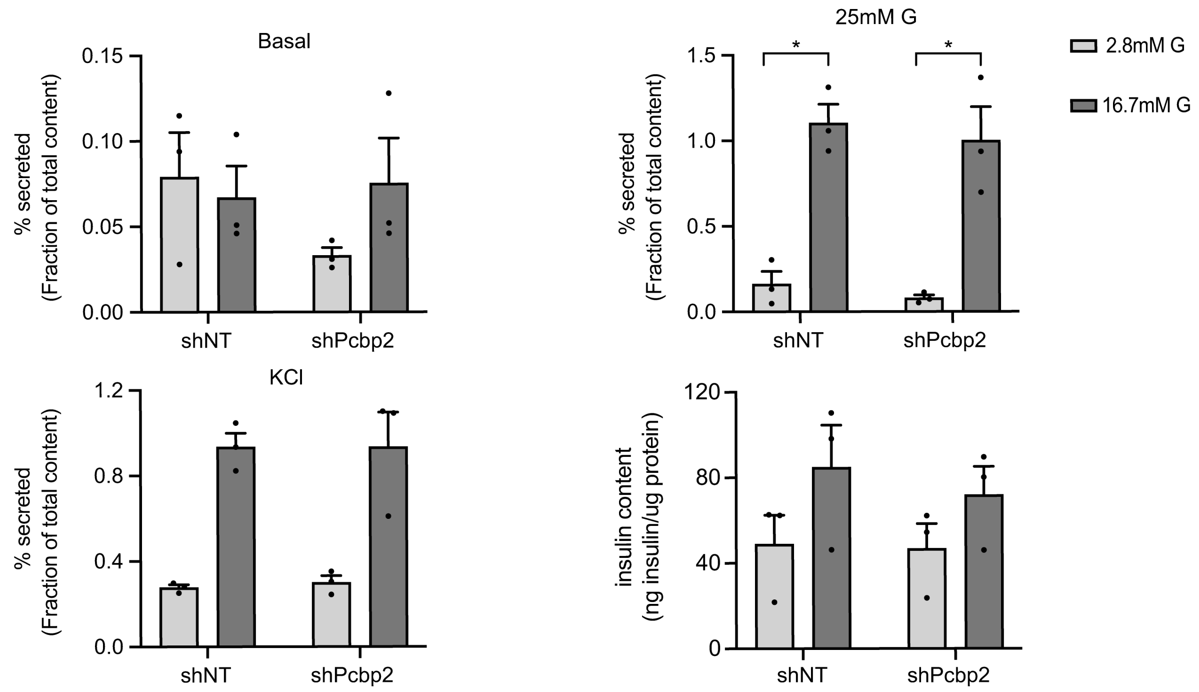

Supplemental Figure 5. Glucose adaptive insulin secretion in response to high glucose and KCl stimulation is maintained during shRNA-mediated *Pcbp2* depletion in β cells. Individual static insulin secretion profiles and insulin content measurements of transduced islets with shRNA targeting non-targeting (shNT) or *Pcbp2* (shPcbp2) pre-incubated with 2.8mM or 16.7mM glucose (n=3).

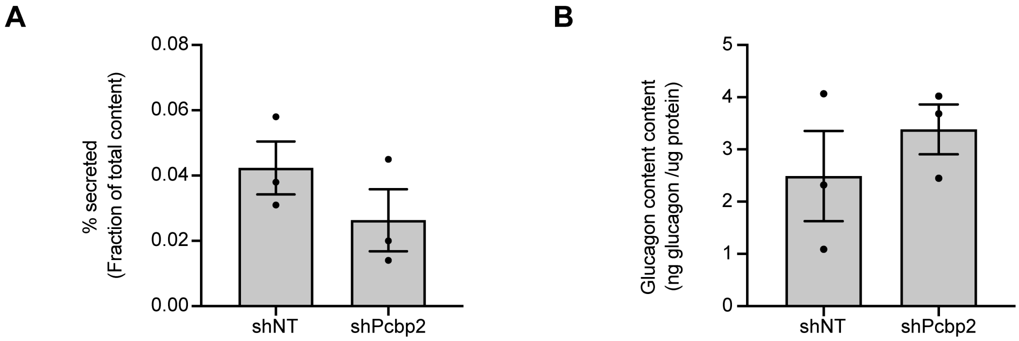

Supplemental Figure 6. IBMX-mediated glucagon secretion is preserved in the setting of shRNA-mediated *Pcbp2* depletion in β cells.
(**A**) Static IBMX-mediated glucagon secretion and (**B**) content of islets transduced with β cell-specific shRNA lentiviral vectors targeting non-targeting (shNT) or *Pcbp2* (shPcbp2) and pre-incubated with 16.7mM glucose.

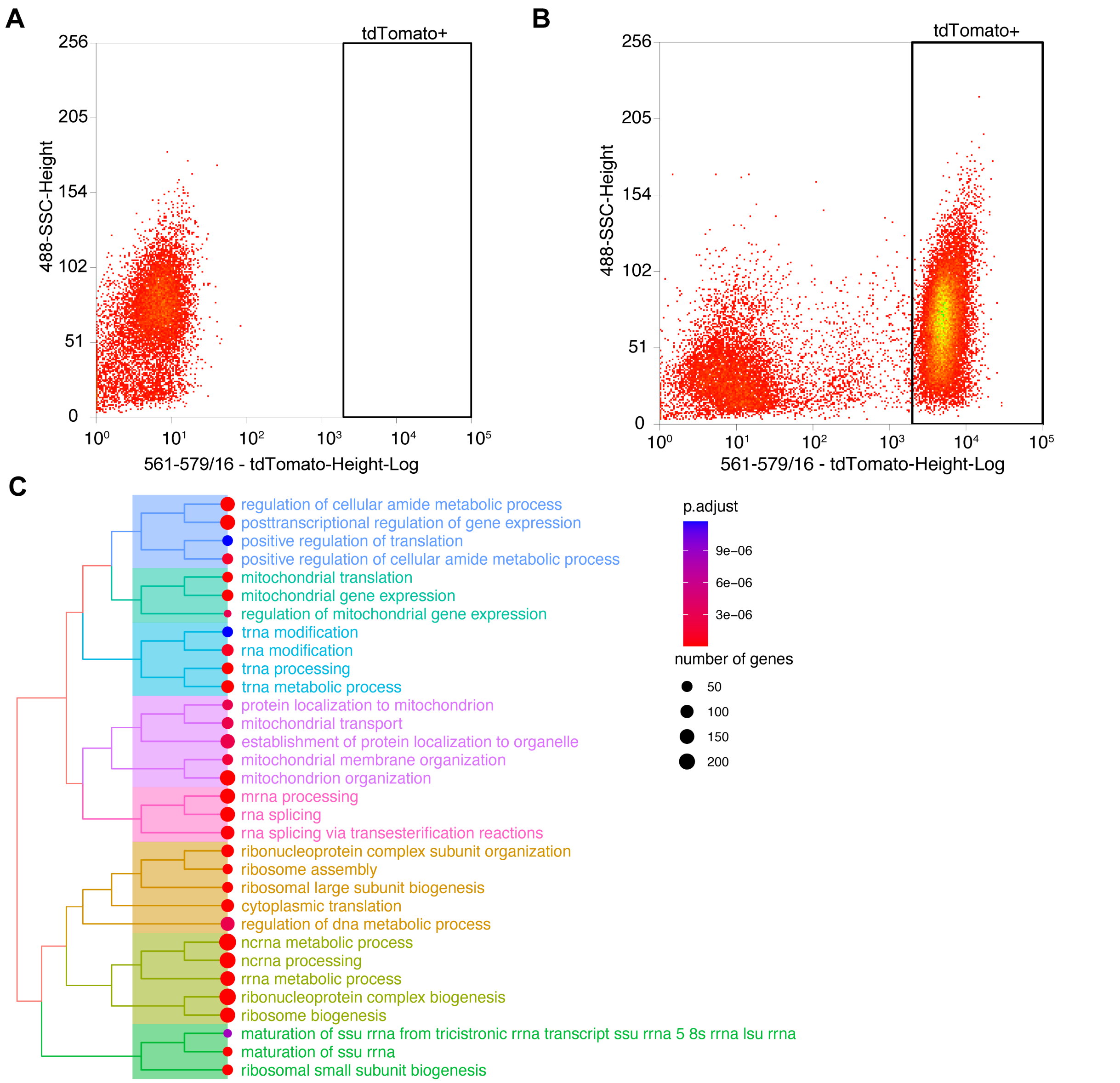

Supplemental Figure 7. A distinct Tdtomato^{+} population is present in *Rosas26^{Tdtomato}* β cells expressing *Cre recombinase,* and high glucose incubation downregulates processes involving RNA processing and post-transcriptional gene regulation. Representative FACS plot for *Rosas26^{Tdtomato}* β cells without (**A**) and with (**B**) Cre recombinase. (**C**) Heatmap plot of terms overrepresented in the gene signatures downregulated in control β cells incubated with high glucose.

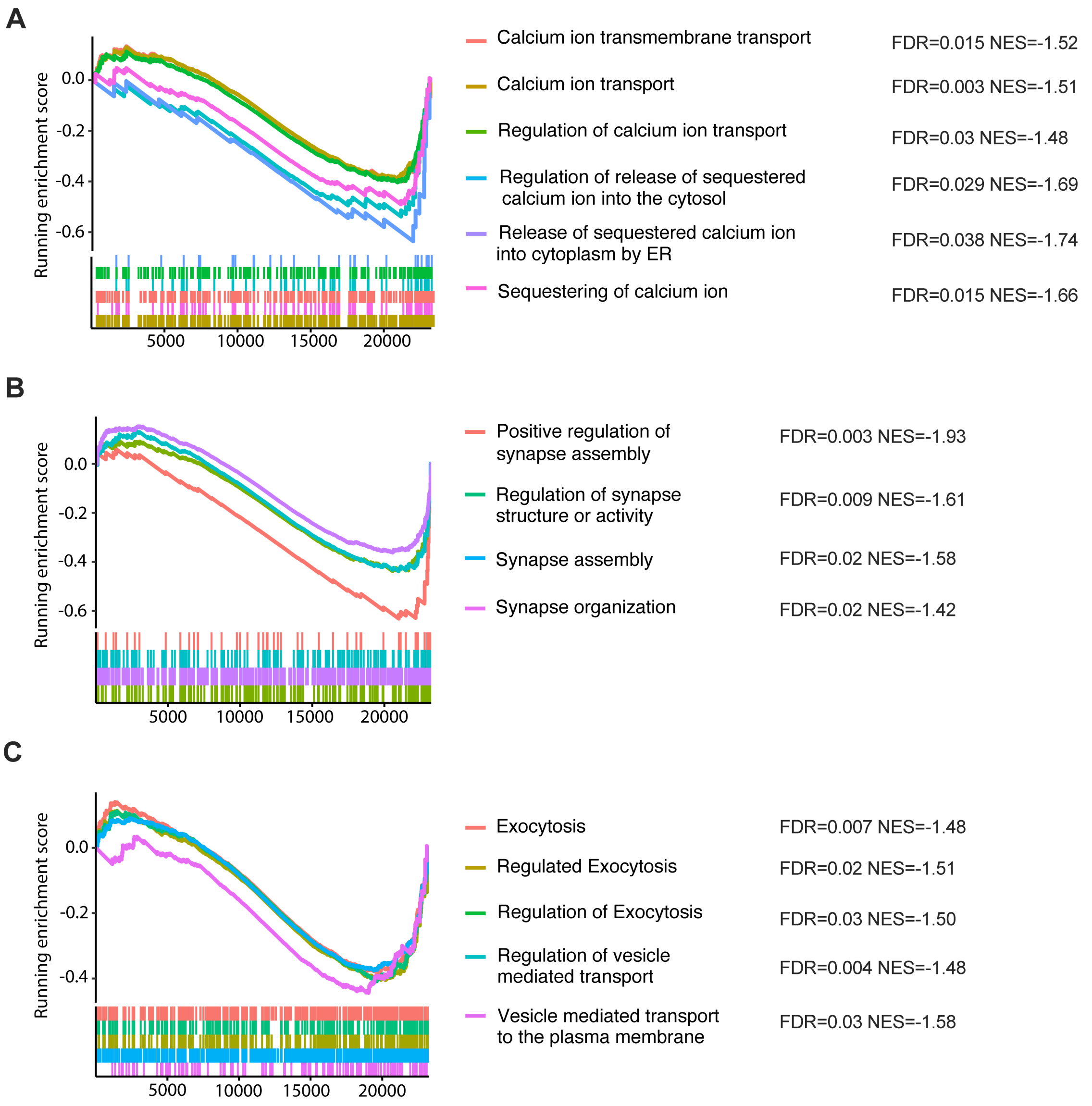

Supplemental Figure 8. Gene sets linked to key aspects of β cell function are downregulated in mutant β cells exposed to sustained high glucose.

GSEA plots showing gene signatures enriched in (**A**) calcium flux, (**B**) synapse assembly and activity, and (**C**) vesicle transport and exocytosis are downregulated in *Pcbp2* deficient β cells during exposure to high glucose.

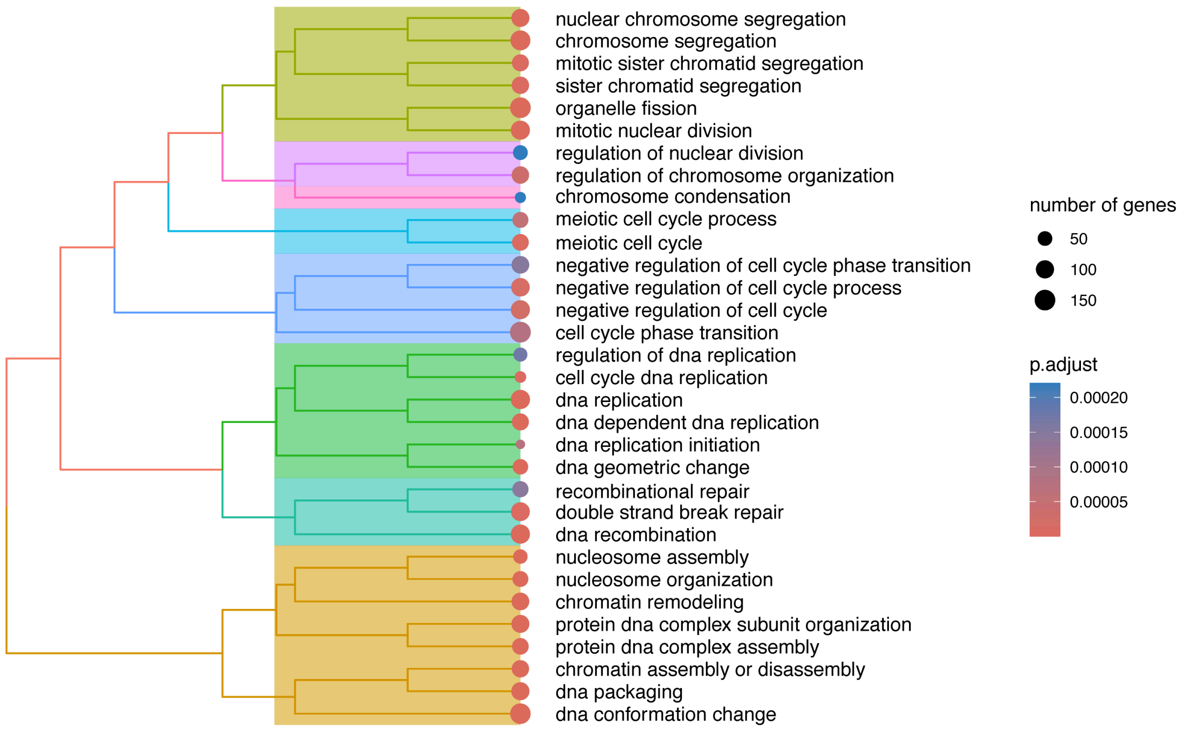

Supplemental Figure 9. Gene sets linked to cell cycle and replication are upregulated in mutant β cells exposed to sustained high glucose.
Heatmap plot of terms overrepresented in the gene signatures upregulated in control β cells stimulated with high glucose.

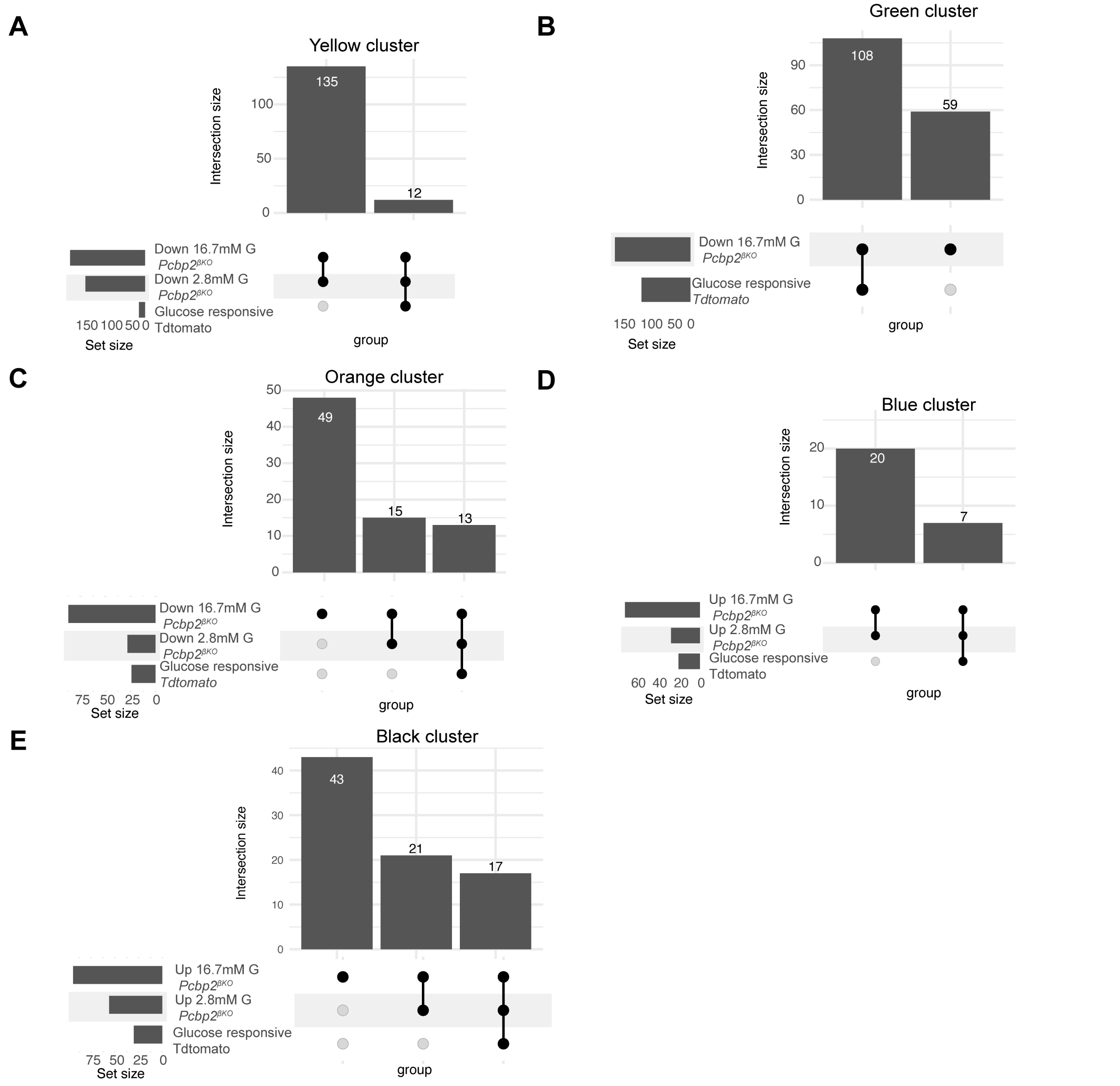

Supplemental Figure 10. Intersecting pairwise differential gene expression comparisons supports the patterns of gene regulation raised from clustering analysis.
(**A**) 73% (135) of yellow cluster genes were unaffected by glucose incubation in control β cells and were reduced under basal and high glucose incubation with *Pcbp2* deficiency. (**B**) 65% (108) of green cluster genes were glucose induced in control β cells and *Pcbp2* deficiency reduced the glucose induction of these genes. (**C**) 56% (49) of genes in orange cluster were unaffected by glucose exposure in control β cells and were uniquely reduced in mutant β cells during high glucose incubation. (**D**) 29% (20) of blue cluster genes were glucose independent in control β cells and were upregulated under basal and high glucose conditions with *Pcbp2* deficiency. (**E**) 45% (43) of black cluster genes were unaffected by glucose stimulation in control β cells and were uniquely upregulated with *Pcbp2* deficiency during high glucose incubation. The horizontal bars in each upset plot set size show the number of genes in the heatmap cluster with differential expression from the noted category. Vertical lines denote the overlapping set of

genes between each category. Vertical barplots display the number of overlapping and distinct sets of genes in each category within each cluster.

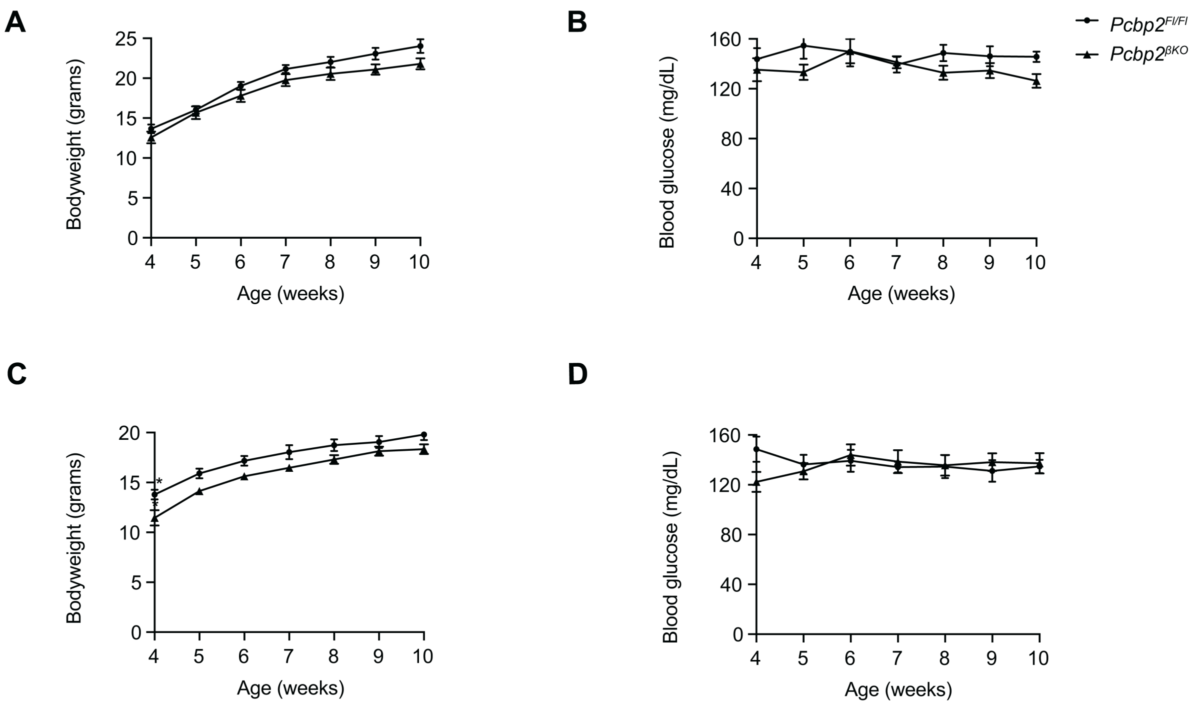

Supplemental Figure 11. ***Pcbp2$^{\beta KO}$*** **mice exhibit normal body weight gain and** ***ad libitum*** **blood glucose levels.**
(**A**) Bodyweight measurements and (**B**) *Ad libitum* blood glucose measurements of male mice (n=8, *Pcbp2$^{Fl/Fl}$*; n=9,*Pcbp2$^{\beta KO}$*). (**C**) Bodyweight measurements and (**D**) *Ad libitum* blood glucose measurements of female mice (n=7, *Pcbp2$^{Fl/Fl}$*; n=8,*Pcbp2$^{\beta KO}$*). *P-value<0.05 by student's 2-tailed t-test.

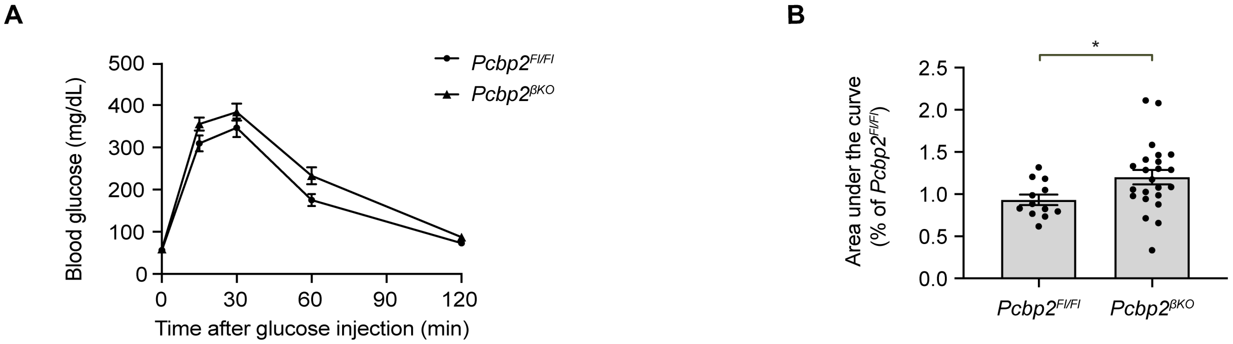

Supplemental Figure 12. Glucose tolerance is less impaired in females.
(**A**) Intraperitoneal glucose tolerance tests performed on 7-8 week old female mice and (**B**) corresponding area under the curve calculations (n=12, *Pcbp2*$^{Fl/Fl}$*;* n=23, *Pcbp2*$^{\beta KO}$). *P-value<0.05 by student's 2-tailed t-test.

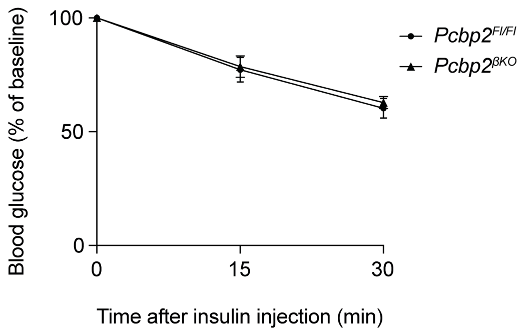

Supplemental Figure 13. Peripheral tissue insulin sensitivity is normal in *Pcbp2$^{\beta KO}$* mice. Insulin tolerance tests performed on 13-15 week old male mice (n=7, *Pcbp2$^{Fl/Fl}$;* n=8, *Pcbp2$^{\beta KO}$*).

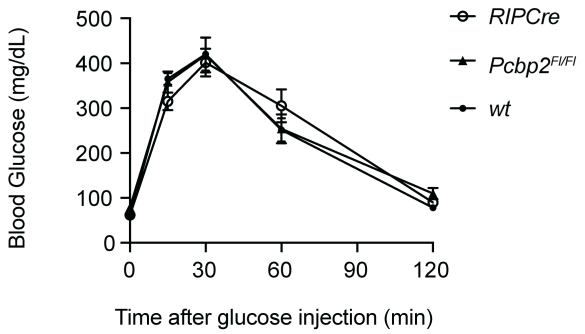

Supplemental Figure 14. *Cre Recombinase* expression and *Pcbp2* loxP site insertion do not influence glucose tolerance.
Intraperitoneal glucose tolerance tests performed on 7-8 week old male mice (n=6, *RIPCre*; n=6, *Pcbp2*$^{Fl/Fl}$*;* n=5, Wildtype).

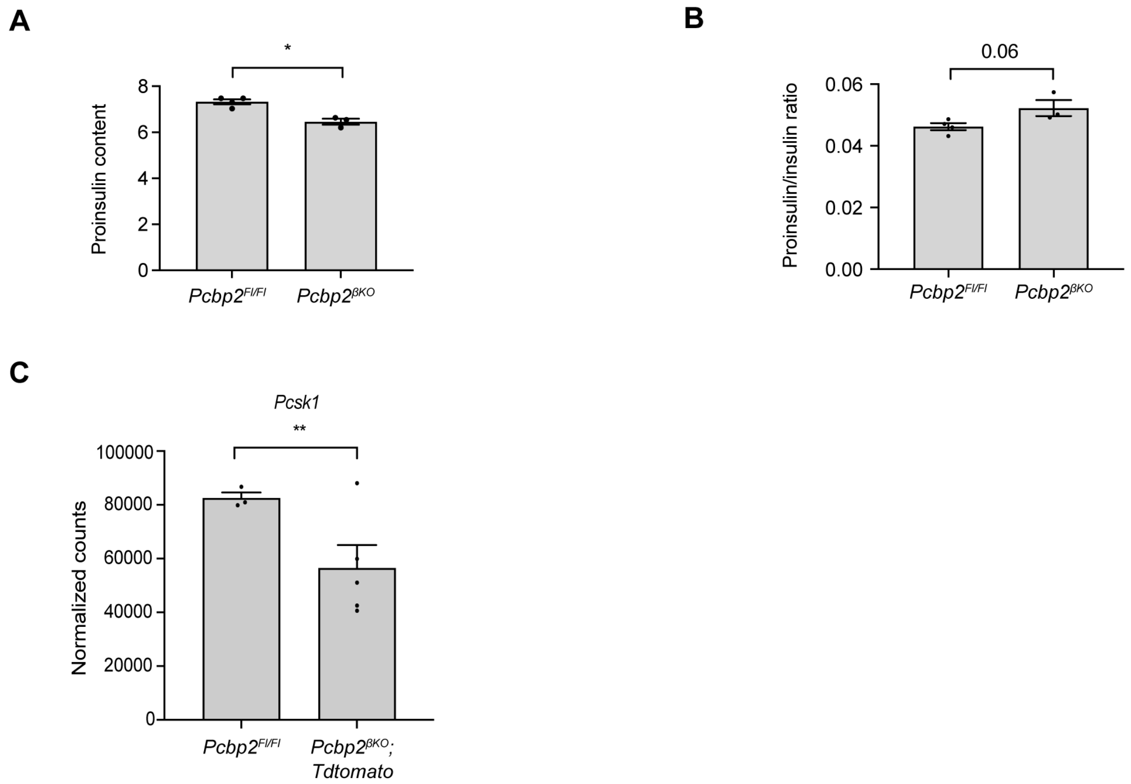

Supplemental Figure 15. *Pcbp2$^{\beta KO}$* islets have defective insulin production. Measurement of pro-insulin content (**A**) and pro-insulin to insulin ratio (**B**) in control and *Pcbp2$^{\beta KO}$* islets from 7-8 week old mice (n=4 *Pcbp2$^{Fl/Fl}$*; n=3 *Pcbp2$^{\beta KO}$*). (**C**) Normalized expression for pro-hormone convertase *Pcsk1* in RNA-sequencing of FACS-sorted control and *Pcbp2* deficient β cells under basal conditions. **P-value<0.01 by student's two-tailed t-test (**A**) or EdgeR differential gene analysis (**C**).

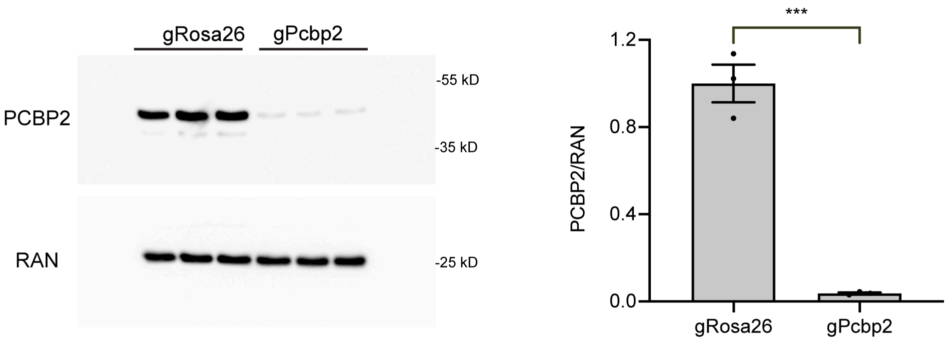

Supplemental Figure 16. Effective CRISPR-mediated depletion of PCBP2.
(**A**) Western blot showing CRISPR-mediated ablation of PCBP2 in Min6 cells (n=3). ***P-value<0.001 by student's two-tailed t-test.

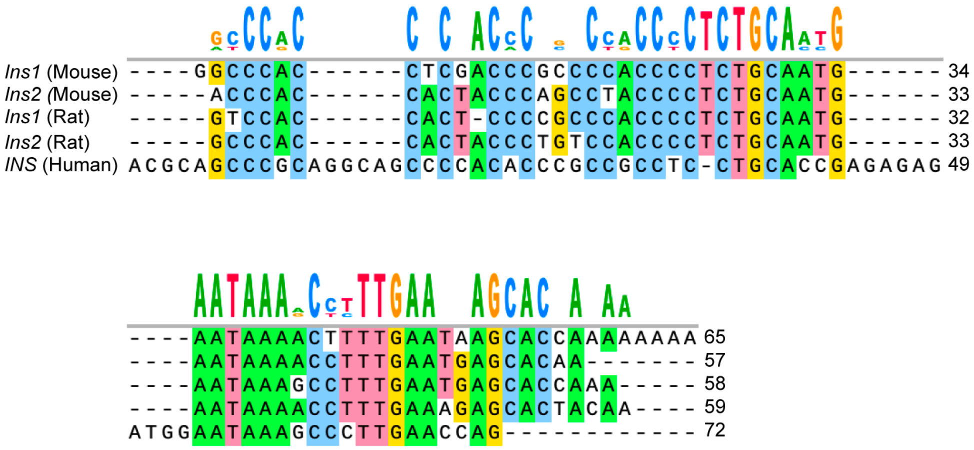

Supplemental Figure 17. the 3’ UTR of insulin harbors a conserved cytosine-rich element. Multiple sequence alignment of the 3’ UTR of rodent and human insulin genes.

A

Pcbp2$^{\beta KO}$* vs *db/db

Rnaset2a	*Gatm*	*Npy*	*Cttnbp2*	*C1qtnf1*
Ncoa4	*Cish*	*Syce2*	*Gabbr2*	*Rapgef5*
H2-Q5	*Cdkn1a*	*Pla2g12a*	*Apob*	*Zcchc12*
Star	*Mmp12*	*Gm11992*	*Atp8a1*	*Retreg1*
Nmnat2	*Zdhhc22*	*Avpr1b*	*St14*	*Chgb*
Ptprd	*Klb*	*Try5*	*Ern1*	*Bcat1*
Edil3	*Socs2*	*Hopx*	*Kcnf1*	*Slc9a9*
Lhfp	*Bsn*	*Cald1*	*Pvr*	*Ivd*
Col19a1	*Kcna2*	*Gpr135*	*Rif1*	*Pon2*
Gipc2	*Atox1*	*Synpr*	*Plod3*	*Tnfrsf11b*
Tph2	*Xdh*	*Col5a2*	*Wipi1*	*Itm2c*
Grem2	*Actn2*	*Runx1*	*Igfals*	*Myo3a*
Pros1	*Kcnk10*	*Cftr*	*Stc1*	*Cd200*
Csf2rb	*Slc41a3*	*Spon1*	*Ppm1e*	*Chn1*
Gm9347	*Adgrd1*	*Slc40a1*	*Kl*	*Enpp2*
Pde7b	*Crispld2*	*Pfkfb4*	*Csrp1*	*Glce*
Rbmx2	*Krt87*	*Rbm3*	*Lgmn*	*Gsdma*
Gdf15	*Gfra3*	*Cux2*	*Nek6*	*Igsf5*
Tmem181c-ps	*Slc23a4*	*Psd4*	*Ogdhl*	*Pecr*
Il34	*Nudt11*	*Adarb2*	*Apobec1*	*Reep5*
Ikzf4	*Adgrg6*	*Adgrf5*	*Palld*	*Sftpd*
A730020E08Rik	*Lrrc8c*	*Mcfd2*	*Car8*	*Mapk4*
Igfbp5	*Myo5c*	*Tubg2*	*Pik3c2g*	*Gsto1*
Fam110c	*Gm43843*	*Lipa*	*Dgkg*	*Slc16a6*
Gbp8	*Car5b*	*Pdzrn3*	*Bex3*	*Nceh1*
Acot9	*AW551984*			

***Pcbp2$^{\beta KO}$* vs T2D**

Rph3a	*Prlr*
Tnfrsf11b	*Grem2*
Mpp1	*Kirrel3*
Slc16a6	*Cpxm2*

***Pcbp2$^{\beta KO}$* vs HG+PA**

Ifrd1	*Gdf15*	*Hopx*	*Gatm*	*Tmem120b*
Kirrel3	*Crem*	*Got1*	*Prlr*	*Tmem97*
Enpp2	*Nefm*	*Cdkn1a*	*Ehhadh*	*Fzd4*
Gtpbp2	*Adm2*	*Pnp*	*Fmo4*	*Cftr*
Wipi1	*Fkbp14*	*Chn1*	*Mgst1*	*Lipa*
Ppm1e	*Ern1*	*Uchl1*	*Rerg*	*Chst9*
Rcbtb2	*Bcat1*	*Kcnj5*	*Galc*	*Reps2*
Gipc2	*St3gal5*	*Pbx1*	*Cmtm8*	*Pde8b*
Trim6	*Pdzrn3*	*Kcna2*	*Marcksl1*	*D630039A03Rik*
Slc15a2				

***Pcbp2$^{\beta KO}$* vs PA**

Akr1b8	*Pfkp*	*Eno1*	*Gdf15*	*Chgb*
Adm2	*Palld*	*Sqstm1*	*Tnfrsf11b*	*Gtpbp2*
Wipi1	*Ern1*	*Galc*	*Lipa*	*Serinc5*
Soat1	*Ivd*	*Ndufs2*	*Rph3a*	*Grem2*
Glce	*Chst9*	*Slc23a2*	*Marcksl1*	*Mpp1*
Vgll4	*Gipc2*	*Rasgrp1*	*Fzd4*	*Chn1*
Nek6	*Matn2*			

B

Ntrk2	*Dgkg*	*Trpm1*	*Syndig1*
Arl15	*Gsto1*	*Ehf*	*Syt10*
Cast	*Soat1*	*Srbd1*	*Adarb2*
Rab3c	*Syce2*	*Zcchc12*	*Adgrg6*
Cttnbp2	*Ern1*	*Myo5c*	*Rasgrp1*
Ptprd	*Kl*	*Enpp3*	*Il34*
Sugct	*Cradd*	*Rapgef5*	*Slc7a7*
Rph3a	*Il13ra1*	*Socs2*	*Dnah8*
Gbp4	*Myo3a*		

Supplemental Figure 18. Genes altered in T2D and/or linked to T2D-associated SNPs overlap PCBP2 regulated genes.

Tables showing (**A**) overlapping genes dysregulated with *Pcbp2* deficiency with those altered in murine and human T2D conditions and (**B**) PCBP2 regulated genes harboring T2D-linked SNPs or mapping to intergenic SNPs associated with T2D.

Supplemental Table 4. Immunofluorescence (IF)/ Western blot (WB) antisera and dilutions

Application	Antigen	Source	Catalog #	Species	Clonality	Concentration
IF	Insulin	DAKO	104840	Guinea Pig	Polyclonal	1:500
IF	Insulin	Proteintech	66198-1-Ig	Mouse	Monoclonal	1:500
IF	PCBP2	Dr. Stephen Liebhaber	N/A	Rabbit	Polyclonal	1:500
IF	mcherry	Proteintech	5f8	Rat	Monoclonal	1:1,000
WB	PCBP2	Dr. Stephen Liebhaber	N/A	Rabbit	Polyclonal	1:5,000
WB	RAN	Proteintech	10469-1-AP	Rabbit	Polyclonal	1:5,000
WB	GAPDH	Cell Signaling	14C10	Rabbit	Monoclonal	1:5,000

Supplementary Table 5. Primer sequences for RT-qPCR.

Gene Name	Forward primer	Reverse primer
Hprt	TGCTCGAGATGTCATGAAGGA	CCAGCAGGTCAGCAAAGAACT
Pcbp2	TAAGAAGATGCGCGAGGAGAG	AAGATGGCATTAGTCGGTCCA
Chgb	TCTGACGGCGGAAGAGAAAAA	AGGCTCGTCTCTCCAACTGT
Ins1	TGGCTTCTTCTACACACCCAAG	ACAATGCCACGCTTCTGCC
Ins2	GCAAGCAGGAAGCCTATCTT	GCTTGACAAAAGCCTGGGTG
Rab3c	GCCCATGCAGATGGCCT	CGTGCTGACGAATGCAGATG
Rph3a	GTAGCCCAGCAGGTTTGAGG	CACTTGGAGGAGCCTCTGTG
Syt10	CCCTTGCTGGAGTTACCTGG	GTGGCCTGGGAGAAGAACAG
