# Supplementary material for: RNA-binding protein PCBP2 regulates pancreatic β cell function and adaptation to glucose
Source: J Clin Invest. 2024 Jun 17;134(12):e172436. doi: 10.1172/JCI172436 (PMC11178539; doi:10.1172/JCI172436)

Full unedited blots for Figure 1A

PCBP2

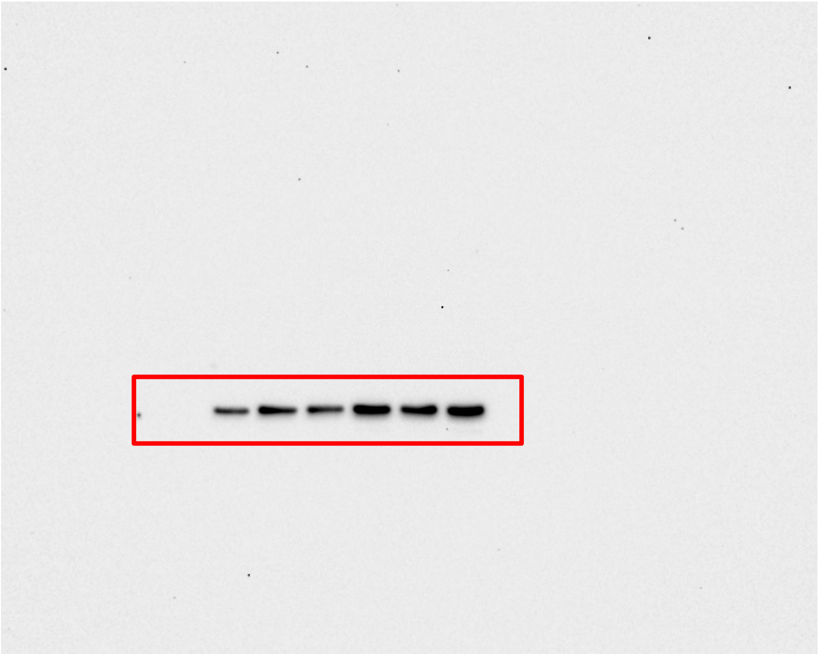

RAN

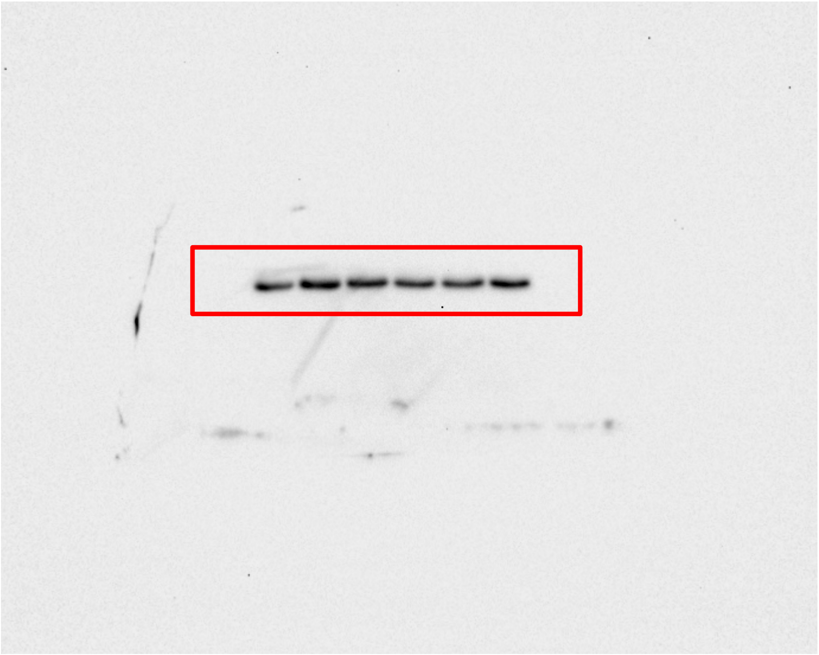

Full unedited blots for Figure 1B

PCBP2

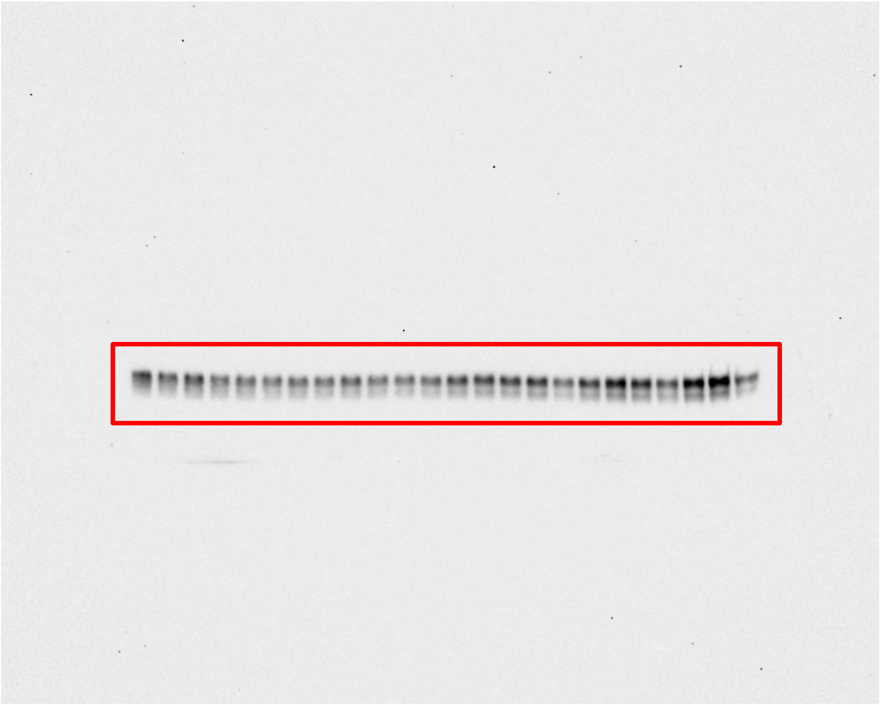

RAN

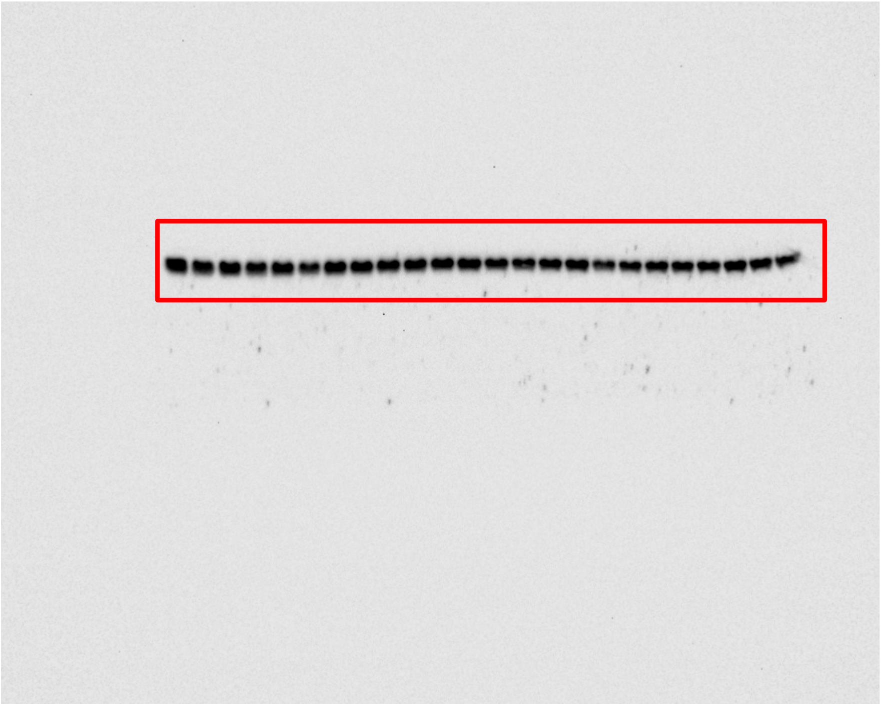

Full unedited blots for Figure 1E

PCBP2

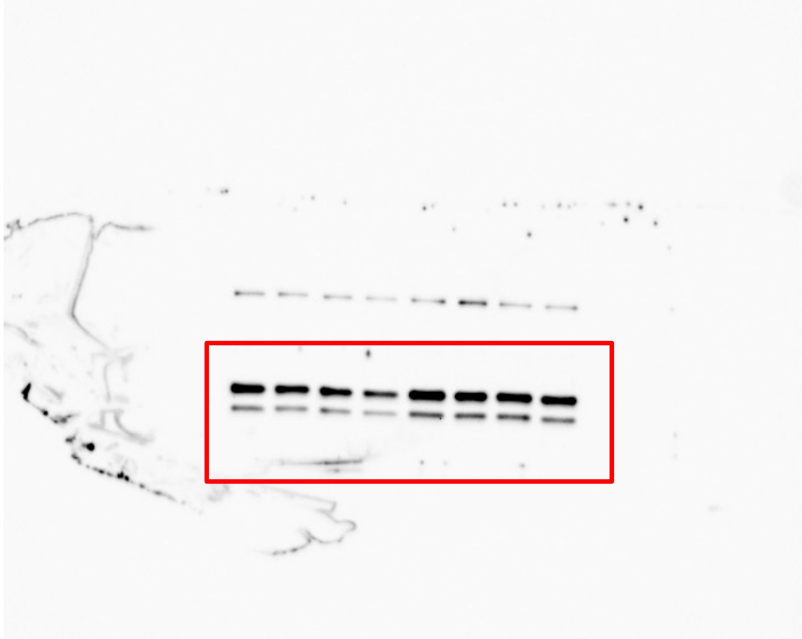

GAPDH

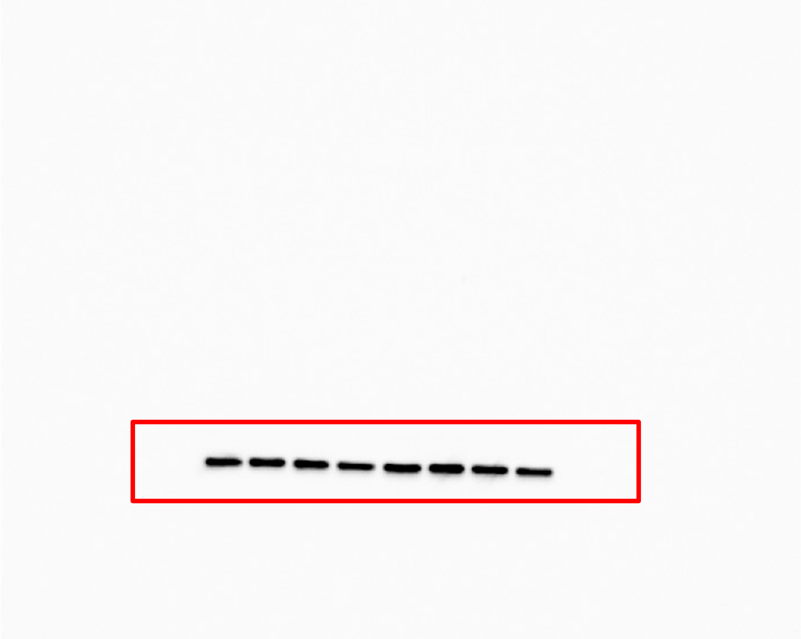

Full unedited blots for Supplemental Figure 1A

PCBP2

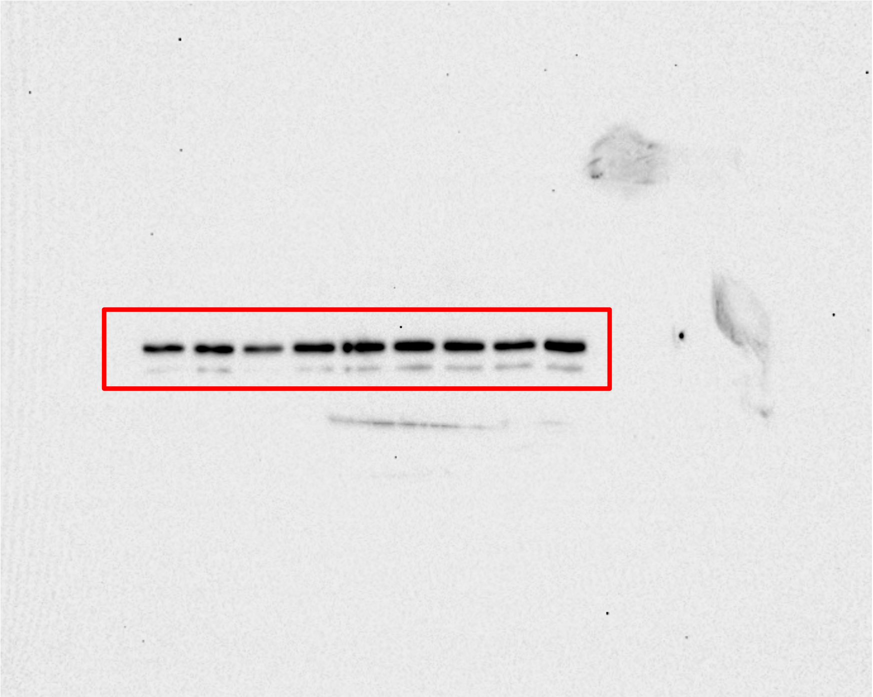

RAN

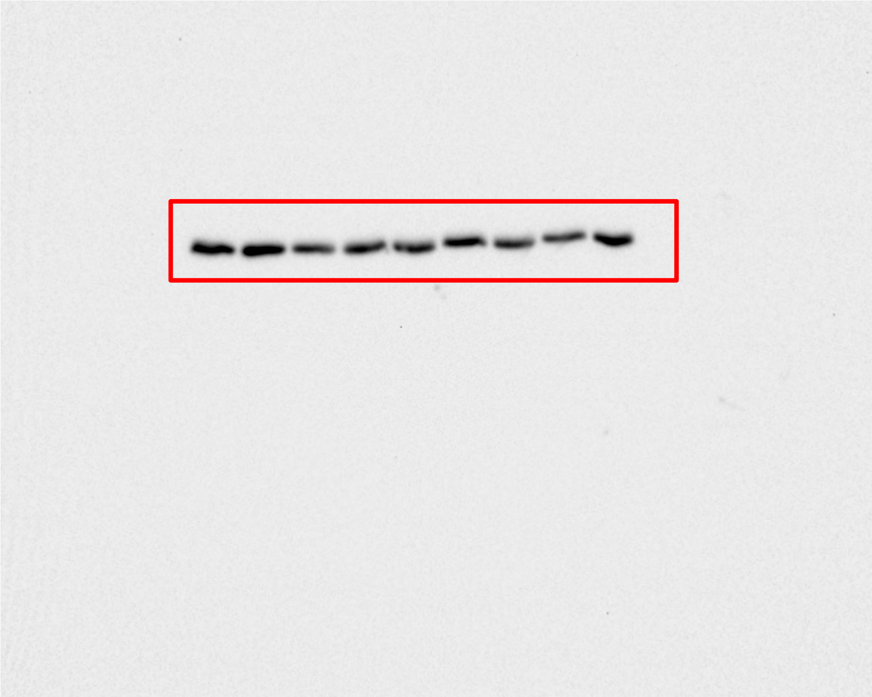

Full unedited blots for Supplemental Figure 1B

PCBP2

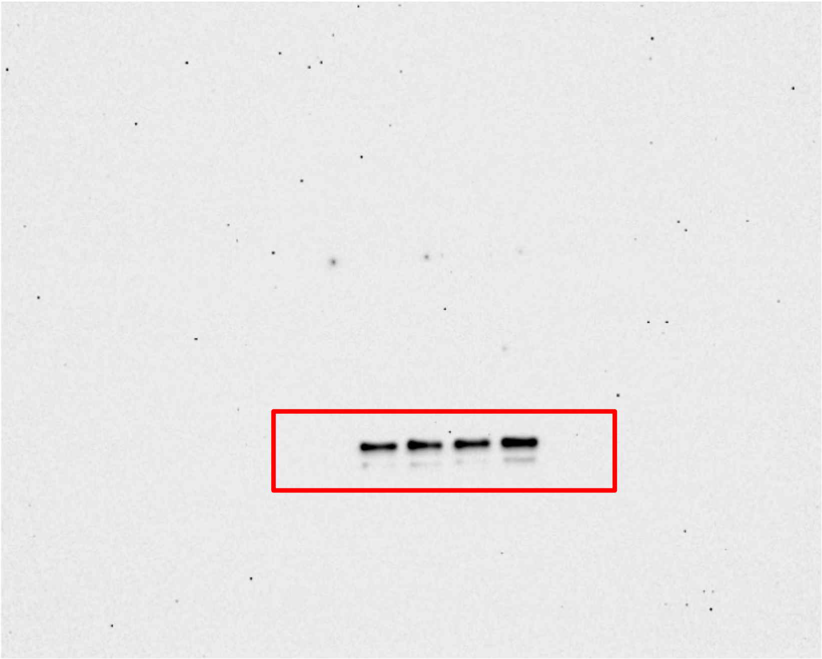

RAN

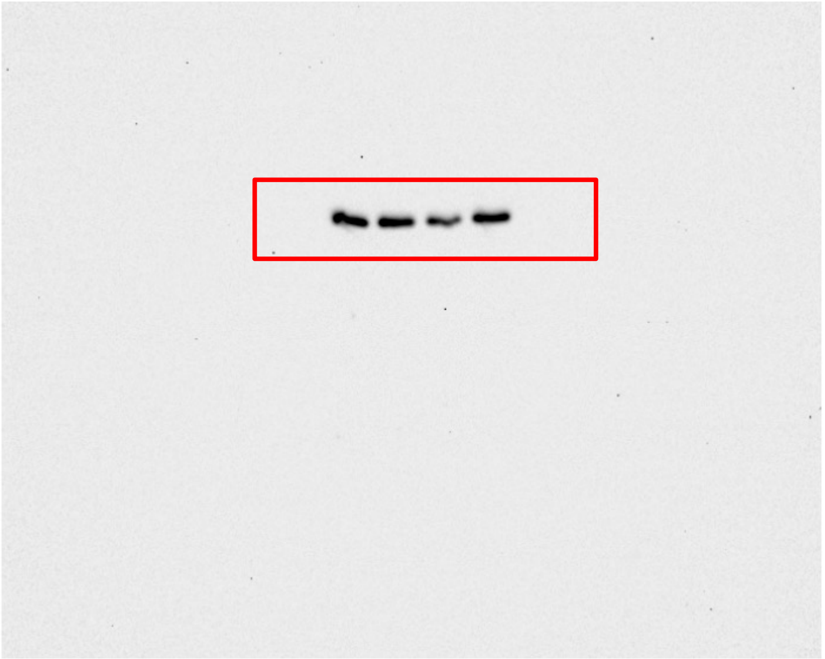

Full unedited blots for Supplemental Figure 4A

PCBP2

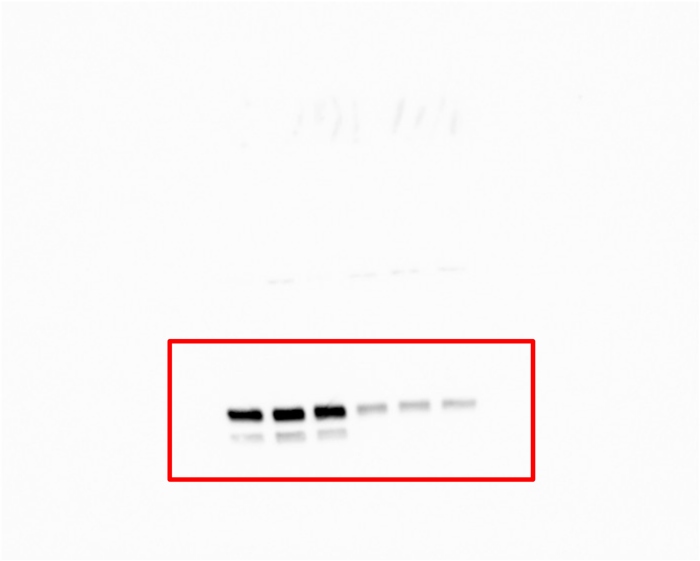

RAN

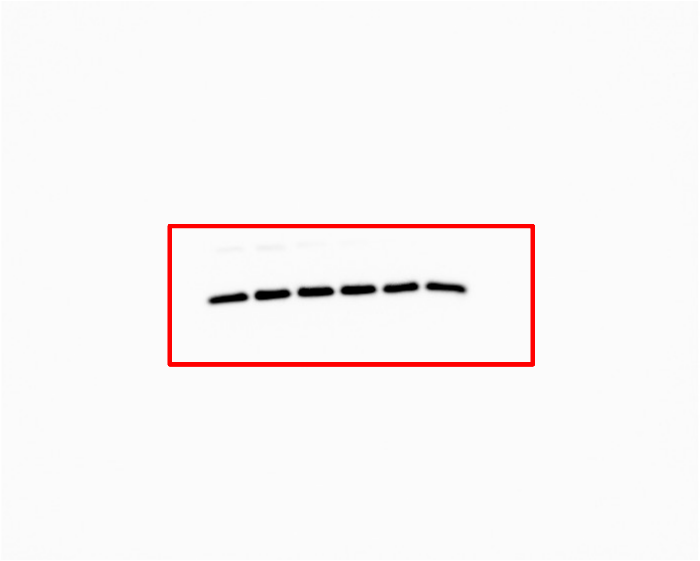

Full unedited blots for Figure 8A

PCBP2

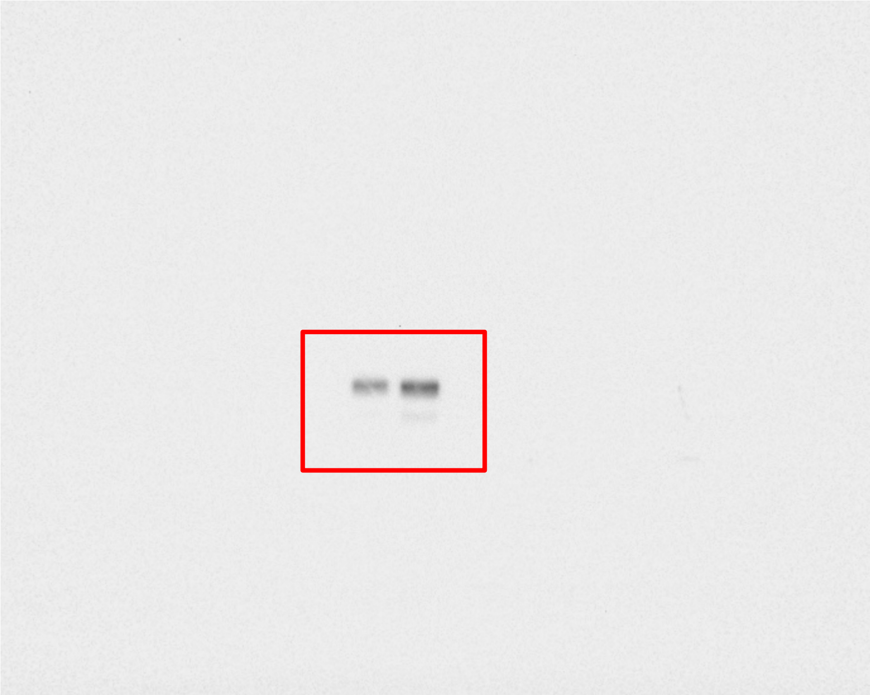

RAN

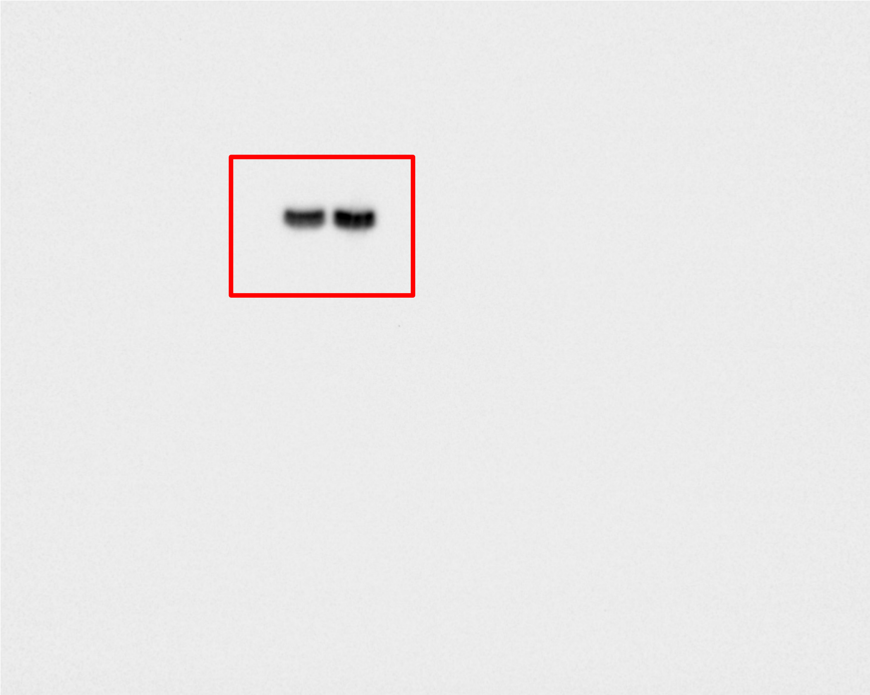

Full unedited blots for Figure 8B

PCBP2

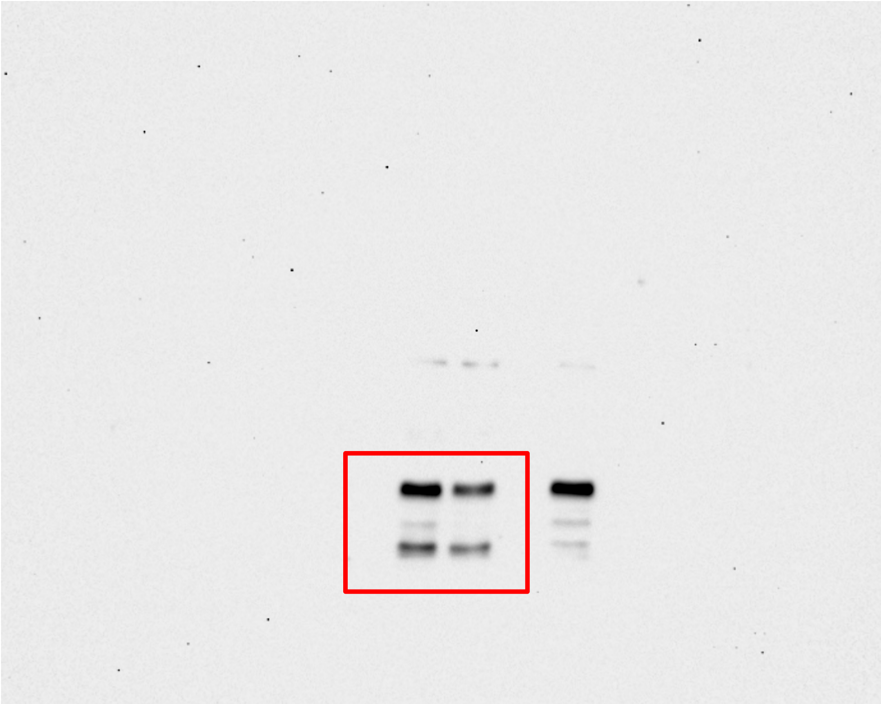

RAN

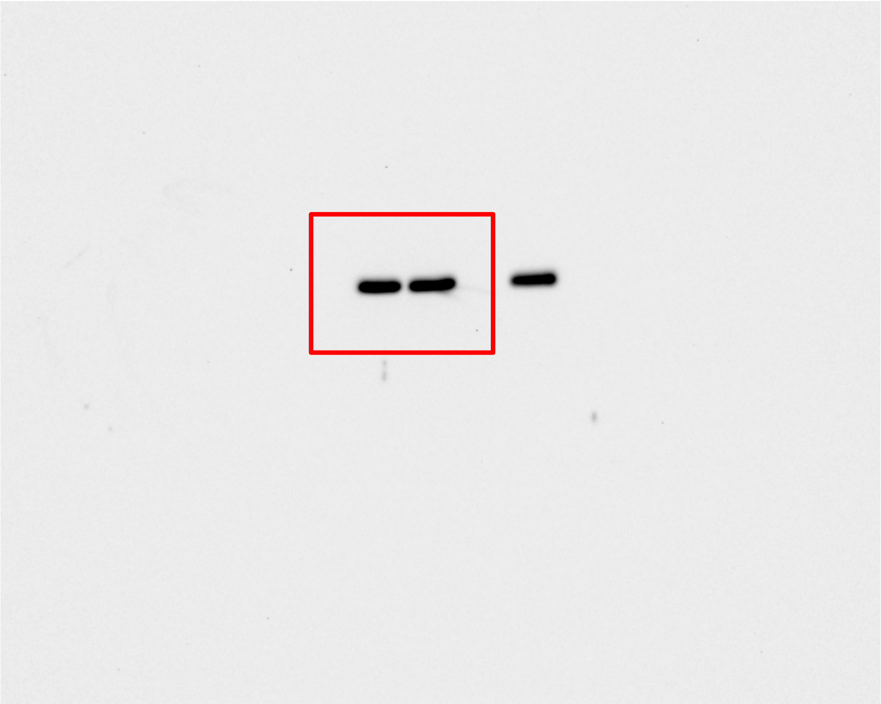

Full unedited blots for Supplemental Figure 14

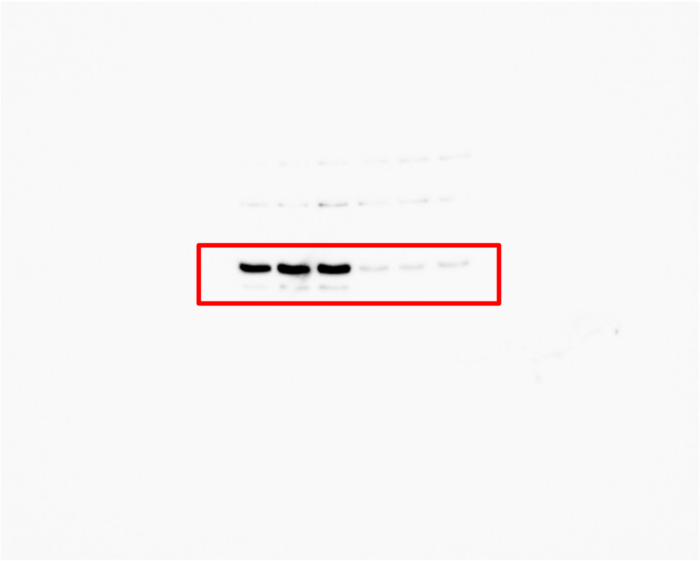

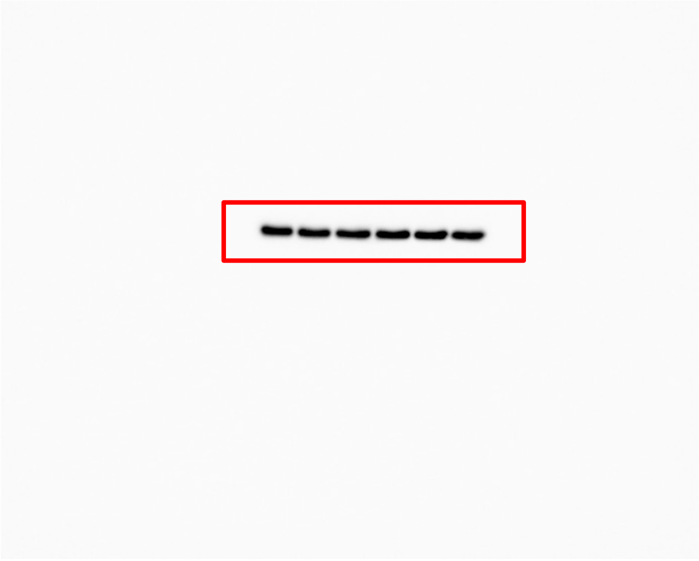

Supplement: Unedited blot and gel images [file jci-134-172436-s011.pdf]
